# Supplementary material for: Prioritization of potential causative genes for schizophrenia in placenta
Source: Nat Commun. 2023 May 15;14:2613. doi: 10.1038/s41467-023-38140-1 (PMC10185564; doi:10.1038/s41467-023-38140-1)
Supplement: Supplementary file 1 — Supplementary Information [file 41467_2023_38140_MOESM1_ESM.pdf]

Supplementary Information for:

Prioritization of potential causative genes for schizophrenia in placenta

Authors: Gianluca Ursini[1,2*], Pasquale Di Carlo[1,3], Sreya Mukherjee[1], Qiang Chen[1], Shizhong Han[1], Jiyoung Kim[1], Maya Deyssenroth[5], Carmen J. Marsit[4], Jia Chen[5], Ke Hao[6], Giovanna Punzi[1,2], Daniel R. Weinberger[1,2,6,7,8,9*].

Affiliations:
[1]Lieber Institute for Brain Development, Johns Hopkins University Medical Campus, Baltimore, Maryland, USA
[2]Department of Psychiatry and Behavioral Sciences, Johns Hopkins University School of Medicine, Baltimore, Maryland, USA
[3]Group of Psychiatric Neuroscience, Department of Basic Medical Sciences, Neuroscience and Sense Organs, University of Bari Aldo Moro, Bari, Italy
[4]Departments of Environmental Health and Epidemiology, Rollins School of Public Health, Emory University, Atlanta, GA
[5]Departments of Environmental Medicine and Public Health, Icahn School of Public Health at Mount Sinai, New York, NY
[6]Department of Genetics and Genomic Sciences, Icahn School of Medicine at Mount Sinai, New York, NY
[7]McKusick-Nathans Institute, Department of Genetic Medicine, Johns Hopkins School of Medicine, Baltimore, MD 21205.
[8]The Solomon H. Snyder Department of Neuroscience, Johns Hopkins School of Medicine, Baltimore, MD 21205.
[9]Department of Neurology, Johns Hopkins School of Medicine, Baltimore, MD 21205.

*Correspondence to:

Gianluca Ursini
Daniel R. Weinberger

855 North Wolfe Street, Suite 300
Lieber Institute for Brain Development,
Johns Hopkins University Medical Campus,
Baltimore, Maryland, USA

Supplementary Note 1: "Selected placental genes potentially causative for schizophrenia".

1. Top-prioritized placenta- schizophrenia- specific risk genes. The 33 top-prioritized genes are here individually highlighted. TWAS-significant association is specific to schizophrenia, and also validated with SMR and colocalization analysis. Among the 33 top-list candidates, we feature the genes as particularly interesting with respect to a possible role in placenta and linked with disease etiology; however, many of these genes have little or are only the subject of more recent literature, thus representing potentially promising novel targets of further functional characterization in placenta. Further experiments are needed to confirm a causative role in affecting brain development and risk for schizophrenia. Gene information is obtained from the scientific literature (references), Isoprofiler Analysis in IPA (Supplementary Data 55b), and NCBI gene database (https://www.ncbi.nlm.nih.gov). Most of these genes have been linked with cancers, consistent with the biological similarity between placental invasion and malignancy[1, 2, 3]. Two top master regulators of 13 (*CD14, CENPM, CRTC2, DDX28, ESAM, GALNT10, GLTP, NRGN, NUDT1, RNF38, RP5_1148A213, RPS17, SLC25A3, TMBIM6)* and 14 *(CD14, CENPM, CRTC2, DDX28, DGCR8, ESAM, GLTP, NRGN, NUDT1, RNF38, RPS17, SLC25A3, TAC3*) of these 33 top-prioritized genes are respectively WNT7 and IL-2R (Supplementary Data 55a), which are known activators of the mTOR pathway[4, 5].

- ***CRTC2* (1q21.3):** alias *TORC2*, the encoded protein is a member of the transducers of regulated cAMP response element-binding protein activity family of transcription coactivators; involved in energy metabolism. Expression of this gene mediates endometrial stromal cells decidualization in humans[6]. Functions associated with this gene include: insulin sensitivity, homeostasis of D-glucose, gluconeogenesis, production of reactive oxidative species (Supplementary Data 55b).
- ***LINC02050* (3p12.2):** lincRNA expressed mostly in testis and placenta. Unknown function.
- ***WDR6* (3p21.31):** this gene encodes a member of the WD-repeat protein family, implicated in cell growth and insulin/IGF-I signaling[7]; a study has also shown its involvement in intellectual disability[8]. It has been associated with cancer (Supplementary Data 55b), again consistent with the biological similarity between placental invasion and malignancy[1, 2].
- ***XXcos-LUCA16.1* (3p21.31):** lncRNA, novel transcript, sense intronic to *GNAI2*. GNAI2 is an alpha subunit of guanine nucleotide binding proteins (G proteins) and is involved in the hormonal regulation of adenylate cyclase.
- ***RP4-555D20.2* (3p21.32):** ncRNA found via an in-silico model as potentially associated with SARS-CoV-2 infection. This gene is located outside GWAS-significant ($p<5e-08$) schizophrenia loci.
- ***RP4-555D20.1* (3p21.32):** Uncategorized gene, To be Experimentally Confirmed (TEC, https://www.gencodegenes.org/pages/biotypes.html). This gene is located outside GWAS-significant ($p<5e-08$) schizophrenia loci.
- ***LINC02484* (4p15.1):** lincRNA, downregulated in human villi at spontaneous abortion in the first trimester[9] ; it has also been associated with hepatocellular carcinogenesis[10], again consistent with the biological similarity between placental invasion and malignancy[1, 2].
- ***CD14* (5q31.3):** this gene encodes a surface antigen that is preferentially expressed on monocytes/macrophages, identified as a target candidate in the treatment of SARS-CoV-2-infected patients to potentially lessen or inhibit inflammation (https://clinicaltrials.gov/ct2/show/study/NCT04391309). Inflammatory functions are mainly associated with this gene (Supplementary Data 55b). In placenta, CD14 is particularly expressed in Hofbauer cells[11], placental macrophages of fetal origin, whose inflammatory responses are induced through Toll-like receptors[12]. It has been associated with the mTOR pathway[13].

- ***GALNT10* (5q33.2):** this gene encodes a glycosylation enzyme and is involved in cancer and energy homeostasis[14]; it has triggered the hypothesis that common genetic variants may alter brain glycosylation in schizophrenia[15]. However, N-Acetylglucosaminyltransferases can also play a role in promoting trophoblast invasion and migration in early placenta[16], and other glycotransferase enzyme have been shown to mediate the relationship between prenatal stress and brain development[17], compatibly with a placental role of this gene linked with schizophrenia risk.
- ***RP3-407E4.3* (6q12):** DENN/MADD domain containing 1C (*DENND1C*) pseudogene. The gene resembled by this pseudogene, *DENND1C*, functions as a guanine nucleotide exchange factor for the early endosomal small GTPase *RAB35*, an oncogene regulating endosomal membrane trafficking, and recently shown to inhibit mTORC1 activity[18].
- ***RP5-1148A21.3* (6q12):** lncRNA, antisense to *PTP4A1*; it has been shown to interact with *miR-545-5p*, in regulating expression of *PTP4A1*, this affecting risk for human ovarian cancer[19]. PTP4A1 is a protein tyrosine phosphatase, highly expressed in syncytiotrophoblasts (STB), villous trophoblasts (VCT), and extravillous trophoblasts (EVT)[11], which can promote proliferation and epithelial-mesenchymal transition via the PI3K/AKT pathway[20].
- ***NUDT1* (7p22.3):** the encoded enzyme (alias MTH1) hydrolyzes oxidized purine nucleoside triphosphates, such as 8-oxo-dGTP, 8-oxo-dATP, 2-hydroxy-dATP, and 2-hydroxy rATP, to monophosphates, thereby preventing misincorporation, which otherwise could cause mutations resulting in carcinogenesis. It has indeed been shown to suppress oxidative damage buildup, caused by excitotoxicity, in both cellular DNA and RNA in the hippocampus, especially in microglia[21]; dependence of glioblastoma cells and stem cells on functional MTH1 launched the protein as a therapeutic target[22]. This gene has indeed been implicated in process linked with cell death, oxidative stress ad tumor (Supplementary Data 55b). It has not been yet studied in placenta, where it may play an important role in oxidative stress response, being expressed in most cell types, particularly in EVT, and in Hofbauer cells and other immune cells[11].
- ***RP4-778K6.3* (7q22.3):** lncRNA transcribed from the same locus of *SRPK2*, a protein kinase that enables ATP binding activity, and magnesium ion binding activity. *SRPK2* is involved in several biological processes, including nucleic acid metabolic process; peptidyl-serine phosphorylation; and regulation of viral genome replication.
- ***RNF38* (9p13.2):** this gene encodes the ring finger protein 38, playing roles in diverse cellular processes including oncogenesis, development, signal transduction, ubiquitination, vesicular transport, and apoptosis (Supplementary Data 55b). It is expressed in various organs, including placenta and brain, where it can be regulated by the serotoninergic signaling[23]. This may be relevant in placenta, given the role of serotonin in mediating the effect of prenatal stress[24]. It has been also associated with the mTOR pathway[25].
- ***MIR4688* (11p11.2):** microRNA expressed in placenta[26]. The host gene is in the same locus of *DGKZ*, highly expressed in EVT[11], whose inhibition has been shown to affect the mTOR signaling[27].
- ***VSIG2* (11q24.2):** a protein-protein interaction with mTOR has been documented for this gene[28] and with subunits of the COG complex (i.e., Conserved Oligomeric Golgi). It has been associated with cancer (Supplementary Data 55b).
- ***ESAM* (11q24.2):** this gene encodes an endothelial adhesion molecule that enables cell-cell adhesion mediator activity, and is involved in several processes, including bicellular tight junction assembly; cell-cell adhesion; and regulation of actin cytoskeleton organization. Located in the cell-cell junction and plasma membrane. Being involved in the blood-brain and intestinal barriers[29], it could also play a role in the blood-placenta barrier. Higher expression of the gene has been associated with birth defects (microcephaly) induced by virus Zika[30]. It has been also identified as

hematopoietic stem cell marker with a role in developing hematopoiesis in fetal liver, affecting mortality of the embryo/infant[31]. It has not yet been studied in placenta, despite high expression of this gene occurs in this organ. It has been associated with the mTOR pathway[32].

- ***ESAM-AS1* (11q24.2):** lncRNA of unknown function, antisense to *ESAM* (see above).
- ***NRGN* (11q24.2):** the encoded protein is target of thyroid hormone in the human brain, where it acts as postsynaptic protein kinase substrate that binds calmodulin in the absence of calcium expression. It may be involved in the consequences of hypothyroidism on mental states, during development as well as in adult subjects. Thyroid hormones are also involved in the formation and functioning of the placenta[33], but the function of *NRGN* in placenta is still unexplored. It also has been associated with the mTOR pathway[34].
- ***TMTC1* (12p11.22):** being involved in protein O-linked mannosylation, *TMTC1* has been proposed to contribute to the development of schizophrenia via brain glycosylation[15], similarly to *GALNT10*. However, this mannosyltransferase[35] has been associated with insulin signaling, cell invasion, epithelial-to-mesenchymal transition (Supplementary Data 55b), all biological processes linked with placenta genomic risk for schizophrenia in our study. It is predicted to be located in endoplasmic reticulum**.**
- ***TMBIM6* (12q13.12):** downregulated in preeclamptic placentae in a endoplasmic reticulum stress model[36]; consistently, the encoded protein, Bax Inhibitor-1, has been shown to protect the neonatal brain after hypoxic-ischemic injury, improving behavioral outcomes in animals[37].
- ***TAC3* (12q13.3):** predominantly expressed in placenta, its circulating placental levels have been studied as biomarkers of late-onset fetal growth restriction[38], and preeclampsia[39]; it is also differentially expressed in pregnant women with Chagas disease[40]. Additionally, this gene is expressed on a primate-specific population of inhibitory neurons in the striatum that emerges early in development[41]. The possible effect of circulating TAC3 of placental origins on the development of these inhibitory neurons has not yet been studied.
- ***SLC25A3* (12q23.1):** this gene encodes a mitochondrial transporter of phosphate and copper[42]; highly expressed in EVT, VCT, and STB[11], it has been linked with mitochondrial deficiencies (Supplementary Data 55b).
- ***RILPL2* (12q24.31):** involved in regulating lysosome morphology; its function has not yet been studied in placenta, where it is expressed in immune cells[11].
- ***GLTP* (12q24.11):** this gene encodes a glycolipid transfer protein, involved in sphingolipid metabolism. Sphingolipids play an important function in regulating placenta development, and its inflammatory response[43]. In placenta, it shows higher expression in STB ad VCT[11].
- ***GPR135* (14q23.1):** this gene encodes an orphan G protein coupled receptor[44]. It enables arrestin family protein binding activity, which is also relevant in regulating trophoblast activity[45]. DNA methylation of this gene in placenta has been associated with maternal smoking[46].
- ***RPS17* (15q25.2):** this gene encodes a ribosomal protein, involved in translation of mRNAs and proteins. Recurrent deletion at this locus has been associated with increased risk of birth defects encompassing cognitive deficits[47]. It is highly expressed in all placental cell types[11], and it has been associated with the mTOR pathway[48].
- ***CTD-2574D22.3* (16p11.2):** TEC, novel transcript, sense intronic to *KCTD13*. KCTD13 enables identical protein binding activity and small GTPase binding activity. It contributes to ubiquitin-protein transferase activity. It is involved in several processes, including cellular protein metabolic process, and negative regulation of Rho protein signal transduction.
- ***DDX28* (16q22.1):** this gene encodes an RNA-dependent ATPase, which is localized in the mitochondria and the nucleus, and can be transported between the mitochondria and the nucleus. It

regulates HIF-2α- and eIF4E2-mediated translation activation in hypoxia[49]. It could therefore play an important role in placenta, where it is more expressed in STB and VCT[11].

- ***CIAO2B* (16q22.1):** involved in chromosome segregation, iron-sulfur cluster assembly and protein maturation by iron-sulfur cluster transfer; mouse placental expression of this gene has been reported decreased in iron deficiency[50].
- ***RP11-818O24.3* (17p13.3):** lncRNA, novel transcript, antisense to *CRK*.
- ***DGCR8* (22q11.21):** the encoded protein forms a complex essential for microRNAs biogenesis, with effects on pregnancy establishment in human[51, 52] and in animal models, where disruption of germ layer specification[53] and morula-to-blastocyst transition[54] has been linked to this gene; associated diseases include the Velocardiofacial Syndrome and DiGeorge Syndrome. DGCR8 is crucial for regulating microRNAs of the C19-MC microRNA cluster, expressed in the placenta, and regulated by imprinting[55, 56, 57]; of note, haploinsufficiency of *DGCR8* has been associated with altered brain development[58]. It has been also associated with the mTOR pathway[59, 60, 61]
- ***CENPM* (22q13.2):** this gene encodes a centromere protein part of the multi-protein complex that binds spindle microtubules to regulate chromosome segregation at cell division; involved in cancer via PI3K/AKT/mTOR signaling pathway[62, 63].

2. Selected prioritized and SMR-validated placenta- schizophrenia- specific risk genes. Selected genes with TWAS-significant association specific to schizophrenia, and also validated with SMR, are here individually highlighted. TWAS-significant association is specific to schizophrenia, and also validated with SMR analysis. Gene information are obtained from scientific literature (references), and NCBI gene database (https://www.ncbi.nlm.nih.gov).

- ***MIR138-1* (3p21.32):** microRNA up-regulated in pre-eclamptic placentae[64]; it improves LPS-induced inflammation and oxidative stress on trophoblasts through targeting RELA and affecting NF-κB signaling[65].
- ***CUL7* (6p21.1):** this gene encodes a component of an E3 ubiquitin-protein ligase complex, which interacts with TP53, CUL9, and FBXW8 proteins. *CUL7* and *FBXW8* expression in trophoblastic cells is regulated via oxygen tension[66], they form a complex with CUL1 that is essential for placenta development[67].
- ***ATP5MGP8* (11p14.1):** pseudogene whose strongest placental eQTL is the functional SNP rs6265 (Vall66Met) in the *BDNF* locus[68]. The gene resembled by this pseudogene, *ATP5MG*, has been linked with immunity-mediated pathways[69] and encodes for a subunit of a mitochondrial ATP synthase that catalyzes ATP synthesis, utilizing an electrochemical gradient of protons across the inner membrane during oxidative phosphorylation.
- ***ATP5F1B* (12q13.3)**: this gene encodes a subunit of mitochondrial ATP synthase. Mitochondrial ATP synthase catalyzes ATP synthesis, utilizing an electrochemical gradient of protons across the inner membrane during oxidative phosphorylation. In cancer, it promotes migration, invasion and proliferation, by activating the FAK/AKT/MMP2 pathway[70].
- ***P2RX4* (12q24.31):** this gene encodes a purinoceptor for ATP, it is activated by extracellular ATP to raise intracellular calcium, thus altering cell signaling. ATP release occurs under pathophysiological, stress and adverse cell conditions, and in particular in preeclampsia. P2RX4 may restore placental cell homeostasis following ATP-induced changes during pathophysiological conditions. Glycosylation seems required for normal functioning of P2RX4[71]. In cancer cells, it has been recently shown that cell death causes ATP release triggering P2RX4 to mediate an mTOR-dependent pro-survival program[72].

- ***CYP19A1* (15q21.2):** this gene encodes a member of the cytochrome P450 superfamily of enzymes, which are monooxygenases catalyzing reactions involved in drug metabolism and synthesis of cholesterol, steroids, and other lipids. This protein localizes to the endoplasmic reticulum and catalyzes the last steps of estrogen biosynthesis. Mutations in the gene can result in either increased or decreased aromatase activity; the associated phenotypes suggest that estrogen functions both as a sex steroid hormone and in growth or differentiation. It is mainly expressed in placenta, where it contributes to hormone synthesis[73].
- ***ATP13A1* (19p13.11):** this gene encodes the P5A-adenosine triphosphatase (ATPase) transporter. P5A-ATPase activity mediates the extraction of mistargeted proteins from the endoplasmic reticulum (ER)[74].
- ***IRF3* (19q13.33):** this gene encodes the interferon regulatory transcription factor 3. The encoded protein is found in an inactive cytoplasmic form that upon serine/threonine phosphorylation forms a complex with CREBBP. This complex translocates to the nucleus and activates the transcription of interferons alpha and beta, as well as other interferon-induced genes. The protein plays an important role in the innate immune response against DNA and RNA viruses, and has been involved in the response to COVID-19, also at the maternal-fetal interface[75]. In placenta, it also plays an important role in the regulation of interferon-beta signaling, which is essential to prevent pregnancy complications[76].
- ***ATF4* (22q13.1):** this gene encodes a transcription factor belonging to a family of DNA-binding proteins (AP-1 family, CREBs, and CREB-like proteins), which share a leucine zipper region that is involved in protein-protein interactions. It is involved in reactive oxidative species-mediated mitophagy signaling in placental trophoblasts[77], and in the unfolded protein response in placenta in response to hypoxia[78].
- ***RP1-85F18.6* (22q13.2):** lncRNA, antisense to *EP300*, with a key role in reduced cell proliferation and invasion, and increased apoptosis and pyroptosis, in cancer[79], and in the regulation of cell cycle[79, 80]. *EP300*, the sense protein-coding gene paired with *RP1-85F18.6*, is an hypoxia-responsive gene[81, 82], whose hypomethylation is associated to prenatal stress[81], and with a role in preeclampsia[83].

3. Selected placenta&brain- pleiotropic- schizophrenia- risk genes. Selected genes with TWAS-significant association specific to schizophrenia, in both placenta and mid-gestational prenatal brain cortex. We hypothesize that genetic variation contributes to risk for schizophrenia also through pleiotropic effects in placenta and fetal brain, regulating expression of some of these genes in both organs. Gene information are obtained from scientific literature (references), and NCBI gene database (https://www.ncbi.nlm.nih.gov).

- ***HSPE1* (2q33.1):** this gene encodes a major heat shock protein which functions as a chaperonin. Heat shock stress response can mediate the effect of prenatal stress in different organs, including placenta and brain[84]. Our TWAS data indicate that excessive activation of the stress response in brain and blunted response in placenta may increase risk for schizophrenia.
- ***STAB1* (3p21.1):** this gene encodes a transmembrane receptor protein which may function in angiogenesis, lymphocyte homing, cell adhesion, or receptor scavenging. *STAB1* is expressed in placental endothelial cells and macrophages during pregnancy, and it may be involved in cell adhesion and molecular scavenging in placental macrophages, affecting placental development and manteinance[85, 86]. In brain, STAB1 contributes to the organization of the dendritic arbor[87].
- ***BAP1* (3p21.1):** this gene encodes the BRCA1 associated protein 1, deubiquitinating enzyme. Downregulation of BAP1 protein is essential to trigger epithelial-mesenchymal transition (EMT)

during trophoblast differentiation, associated with a gain of invasiveness[88]. The function of BAP1 in brain is not yet well characterized; however, because of its interaction with α-Synuclein, it may play a role in synaptic vesicle trafficking and subsequent neurotransmitter release[89]. During hypoxia, BAP1 binds, deubiquitylates, and stabilizes HIF-1α, the master regulator of the hypoxia response[90].

- ***FXR1* (3q26.33):** this gene encodes an RNA binding protein with an important role in protein translation. Its function in brain has been associated with homeostatic plasticity, response to sleep deprivation[91], emotion processing[92], modulation of glutamatergic transmission[93]. In placenta, *FXR1* expression is decreased in trophoblasts from patients with unexplained recurrent spontaneous abortion; consistently, FXR1 promotes trophoblast migration at early pregnancy[94]. It is therefore possible that genetic variation affecting expression in both placenta and brain may impair placenta development, and therefore brain development, and – also independently – brain function, thus increasing risk for schizophrenia.
- ***GAB1* (4q31.21):** this gene encodes a member of the IRS1-like multisubstrate docking protein family. It is an important mediator of branching tubulogenesis and plays a central role in cellular growth response, transformation and apoptosis. Maternally-imprinted specifically in mouse placenta, but not in humans[95], it is downregulated in preeclampsia, while its upregulation by EGR1 contributes to trophoblast cell proliferation and invasion[96]. In brain, it mediates PDGF signaling and interact with GSK3B and beta-catenin to promote oligodendrocyte differentiation and CNS myelination[97]; it is also expressed in excitatory neurons, where it interacts with SHP2 (encoded by *PTPN11*), leading to ERK activation[98].
- ***TRIM8*** and ***TRIM8-DT*** **(10q24.32):** this gene encodes the human tripartite motif containing protein 8 (or RING finger protein 27); this gene and its divergent transcript show, in our analysis, a strong negative association with schizophrenia in both placenta and brain. TRIM8 has been defined as a molecule of duality, as it can have either anti-proliferative (linked with Tp53) or oncogenic activity (linked with NfKb), depending also on cellular or physiological conditions, and exposure to stress[99]. In neurons, it has been also shown to play a role in the response to oxygen-glucose deprivation/re-oxygenation[100].
- ***EIF5* (14q32.32):** this gene encodes the eukaryotic translation initiation factor-5 forming a ribosomal initiation complex that is active in peptidyl transfer and chain elongations. Being involved in the initiation step of protein translation, it is linked to the phosphatidylinositol-3-kinases PI3K/AKT/mTOR pathway[101]. It has been proposed to play a role in preeclampsia together with other genes in human accelerated regions and under positive selection[102]. Of note, our data show that schizophrenia risk is associated with i) predicted up-regulation of EIF5 in brain, compatibly with the possibility that a dysregulation of pathways linked with protein translation is detrimental for brain development[103], and ii) down-regulation in placenta, consistent with the crucial role of EIF[104] and mTOR[105] signaling in placenta development.
- ***FURIN* (15q26.1):** this gene encodes a member of the subtilisin-like proprotein convertase family, which includes proteases that process protein and peptide precursors trafficking through regulated or constitutive branches of the secretory pathway. Some of its substrates include proparathyroid hormone, transforming growth factor beta 1 precursor, proalbumin, pro-beta-secretase, membrane type-1 matrix metalloproteinase, beta subunit of pro-nerve growth factor and von Willebrand factor. The spike protein of SARS-CoV-2 is thought to be uniquely cleaved by this protease. While an effect of a common variant in *FURIN* on synaptic function has been shown in cellular models[106], this gene is also expressed in placenta, where it could play a role in syncytialization[107], and in trophoblast migration and invasion[108].

- ***MAPK3* (16p11.2):** this gene encodes a MAP kinase, also known as extracellular signal-regulated kinases (ERKs), acting in a signaling cascade that regulates various cellular processes such as proliferation, differentiation, and cell cycle progression in response to a variety of extracellular signals. Alteration of its gene expression in brain has been linked with schizophrenia and neurodevelopmental phenotypes[109]. However, this pathway is also essential for trophoblast growth and invasiveness[110].
- ***SREBP2* (22q13.2):** this gene encodes a a transcription factor that controls cholesterol homeostasis by regulating transcription of sterol-regulated genes. Placental expression of this gene is sensitive to maternal vitamin and mineral supplementation[111]; it can also contribute to mediate the effect of cholesterol oxidation as a determinant of trophoblast function and activity[112] and, more in general, its expressions in placenta is affected by changes in maternal metabolism that may subsequently affect fetal development[113]. In brain, it regulates oligodendrocyte myelination[114]; moreover, glial SREBP2 regulates cholesterol homeostasis, which is critical for neuronal function[115].

Supplementary Note 2: "TWAS replication"

We performed a replication TWAS to further test the hypothesis that the placental transcriptome mediates in part genomic risk for schizophrenia. To this purpose, we leveraged transcriptomic and genomic data from 70 placentae (see Supplementary Data 62 for sample info) from the Eunice Kennedy Shriver National Institute of Child Health and Human Development (NICHD) Fetal Growth Study[116] (dbGaP accession: phs001717.v1.p1). The related raw sequencing data were not publicly available, so that we could not use our RNAseq processing pipeline to obtain expression data at gene and transcript level. We therefore used for TWAS replication the gene expression data available as FPKM on the dbGaP website.

In the whole dataset (N=70), we detected 4850 genes with heritable gene expression, 3535 of which overlapped the 8558 genes with heritable cis-regulated expression in our discovery sample. 57 of these 3535 genes were among the 187 TWAS-significant genes in the analyses in our whole discovery sample (Supplementary Data 63). The TWAS association was replicated ($p<0.05$) for 21 out of these 57 genes with the same directionality (i.e., Z-score sign), thus showing a level of replication higher than expected by chance (χ^2 with Yates correction = 29.79, $p<0.0001$). Consistently, we found a significant positive (Supplementary Fig. 3a) and concordant (Supplementary Fig. 3b) association between the Z scores from the two TWAS analyses in the discovery and in the replication samples (relationship between Z scores: t= 23.40, r=0.36, Rho=0.40, p<2e-16; Supplementary Fig. 3a,b). The correlation was stronger when considering only TWAS-significant genes in the discovery sample (r=0.51, Rho=0.60, p=5.02e-05; Supplementary Fig. 3a).

In the female dataset (N=35), we detected 5363 genes with heritable gene expression, 3856 of which overlapped the genes with heritable cis-regulated expression in our discovery sample. 49 out of these 3856 genes were among the 225 TWAS-significant unique genes in the analyses in our female discovery sample (Supplementary Data 64). The TWAS association was replicated ($p<0.05$) for 13 out of these 49 genes with the same directionality (i.e., Z-score sign), thus showing a level of replication higher than expected by chance (χ^2 with Yates correction = 7.82, p=0.005). Consistently, we found a significant positive (Supplementary Fig. 3c) and concordant (Supplementary Fig. 3d) association between the Z scores from the two TWAS analyses in the discovery and in the replication samples (relationship between Z scores: t= 22.24, r=0.34, Rho=0.39, p<2e-16; Supplementary Fig. 3c,d). The correlation was slightly stronger when considering only TWAS-significant genes in the discovery sample (r=0.40, Rho=0.42, p=0.0025; Supplementary Fig. 3c).

In the male dataset (N=35), we detected 5299 genes with heritable gene expression, 3764 of which overlapped the genes with heritable cis-regulated expression in our discovery sample. 43 out of these 3764 genes were among the 215 TWAS-significant unique genes in the analyses in our male discovery sample (Supplementary Data 65). The TWAS association was replicated ($p<0.05$) for 22 out of these 43 genes with the same directionality (i.e., Z-score sign), thus showing a level of replication higher than expected by chance (χ^2 with Yates correction = 61.02, $p<0.0001$). Consistently, we found a significant positive (Supplementary Fig. 3e) and concordant (Supplementary Fig. 3f) association between the Z scores from the two TWAS analyses in the discovery and in the replication samples (relationship between Z scores: t= 24.24, r=0.37, Rho=0.40, p<2e-16; Supplementary Fig. 3e,f). The correlation was stronger when considering only TWAS-significant genes in the discovery sample (r=0.73, Rho=0.65, p=3.2e-08; Supplementary Fig. 3e).

Given the limited sample size of this dataset, we did not use these results to further prioritize placental genes with a potential causative role on risk for schizophrenia. However, considered as a whole, these analyses support the reliability of our findings and the relevance of the placental transcriptome in mediating the effect of genomic risk for schizophrenia.

Supplementary Figures

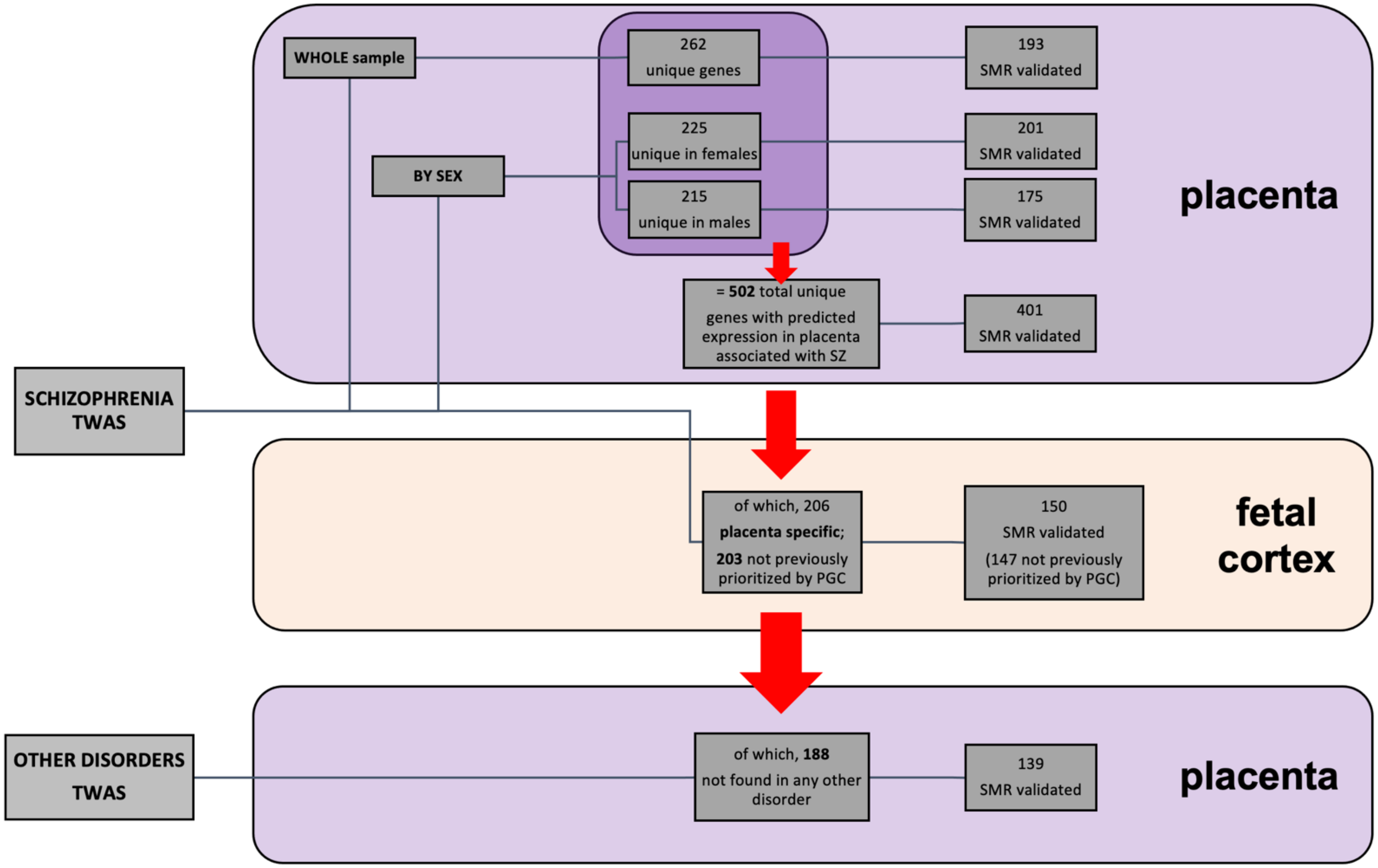

Supplementary Fig. 1: Workflow for the prioritization of the placenta and schizophrenia-specific risk genes.

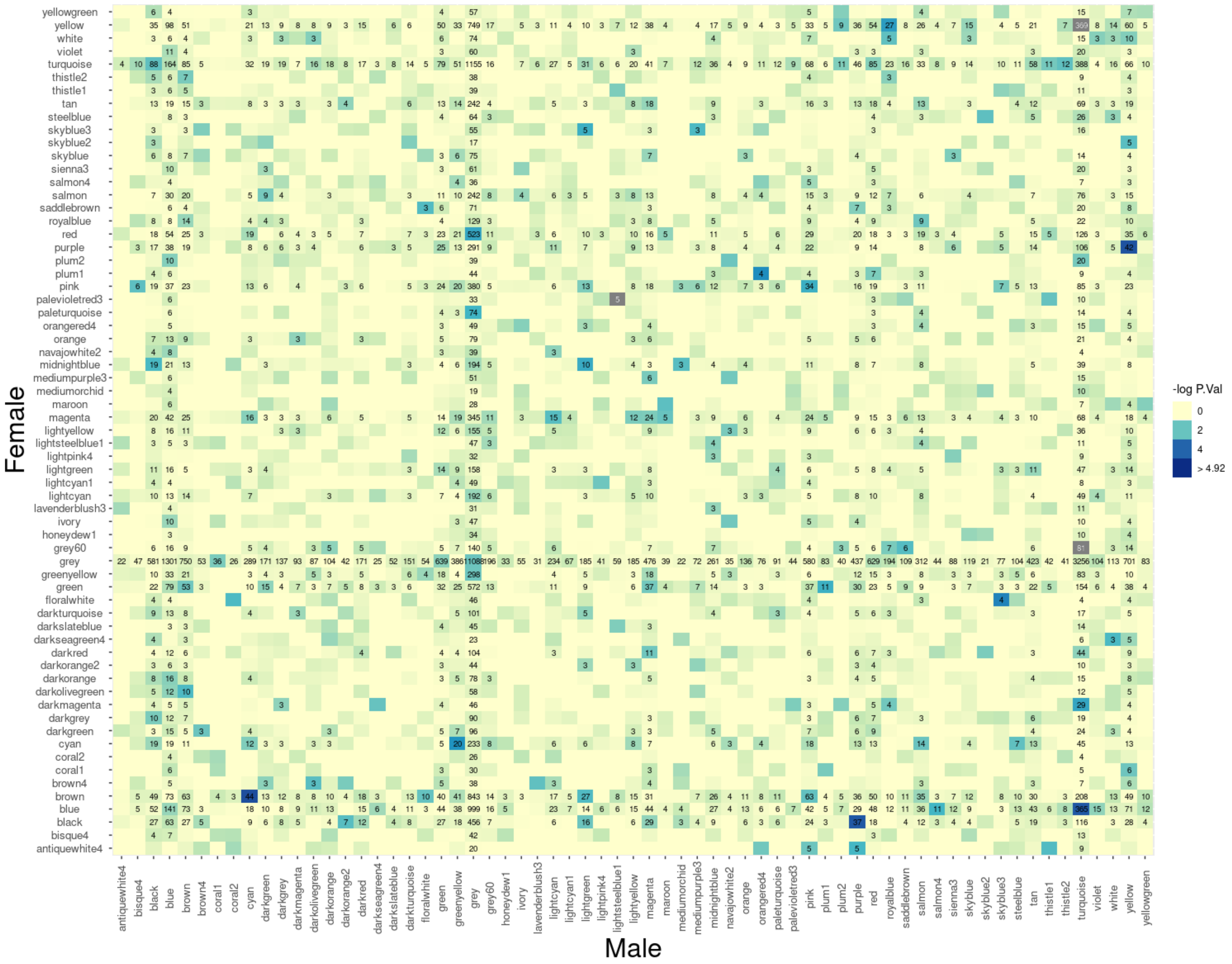
Female
yellowgreen
yellow
white
violet
turquoise
thistle2
thistle1
tan
steelblue
skyblue3
skyblue2
skyblue
sienna3
salmon4
salmon
saddlebrown
royalblue
red
purple
plum2
plum1
pink
palevioletred3
paleturquoise
orangered4
orange
navajowhite2
midnightblue
mediumpurple3
mediumorchid
maroon
magenta
lightyellow
lightsteelblue1
lightpink4
lightgreen
lightcyan1
lightcyan
lavenderblush3
ivory
honeydew1
grey60
grey
greenyellow
green
floralwhite
darkturquoise
darkslateblue
darkseagreen4
darkred
darkorange2
darkorange
darkolivegreen
darkmagenta
darkgrey
darkgreen
cyan
coral2
coral1
brown4
brown
blue
black
bisque4
antiquewhite4
antiquewhite4
bisque4
black
blue
brown
brown4
coral1
coral2
cyan
darkgreen
darkgrey
darkmagenta
darkolivegreen
darkorange
darkorange2
darkred
darkseagreen4
darkslateblue
darkturquoise
floralwhite
green
greenyellow
grey
grey60
honeydew1
ivory
lavenderblush3
lightcyan
lightcyan1
lightgreen
lightpink4
lightsteelblue1
lightyellow
magenta
maroon
mediumorchid
mediumpurple3
midnightblue
navajowhite2
orange
orangered4
paleturquoise
palevioletred3
pink
plum1
plum2
purple
red
royalblue
saddlebrown
salmon
salmon4
sienna3
skyblue
skyblue2
skyblue3
steelblue
tan
thistle1
thistle2
turquoise
violet
white
yellow
yellowgreen
Male
-log P.Val
0
2
4
> 4.92

Supplementary Fig. 2: Heatmap of the overlap between transcript coexpression modules in male (columns) and female (rows) placentae. Color bar represents the significance of the overlap in gene transcript composition (one-sided Fisher exact-test p-value; -log10 p-value 4.92 corresponds to the p=0.05 after Bonferroni-correction) between modules from the WGCNA analyses performed in female (N=73) and male (N=74) placentae. Numbers in each square indicate the count of transcripts shared by each pair of modules (absence of number indicate overlap < 3 transcripts). See also Supplementary Data 25,26.

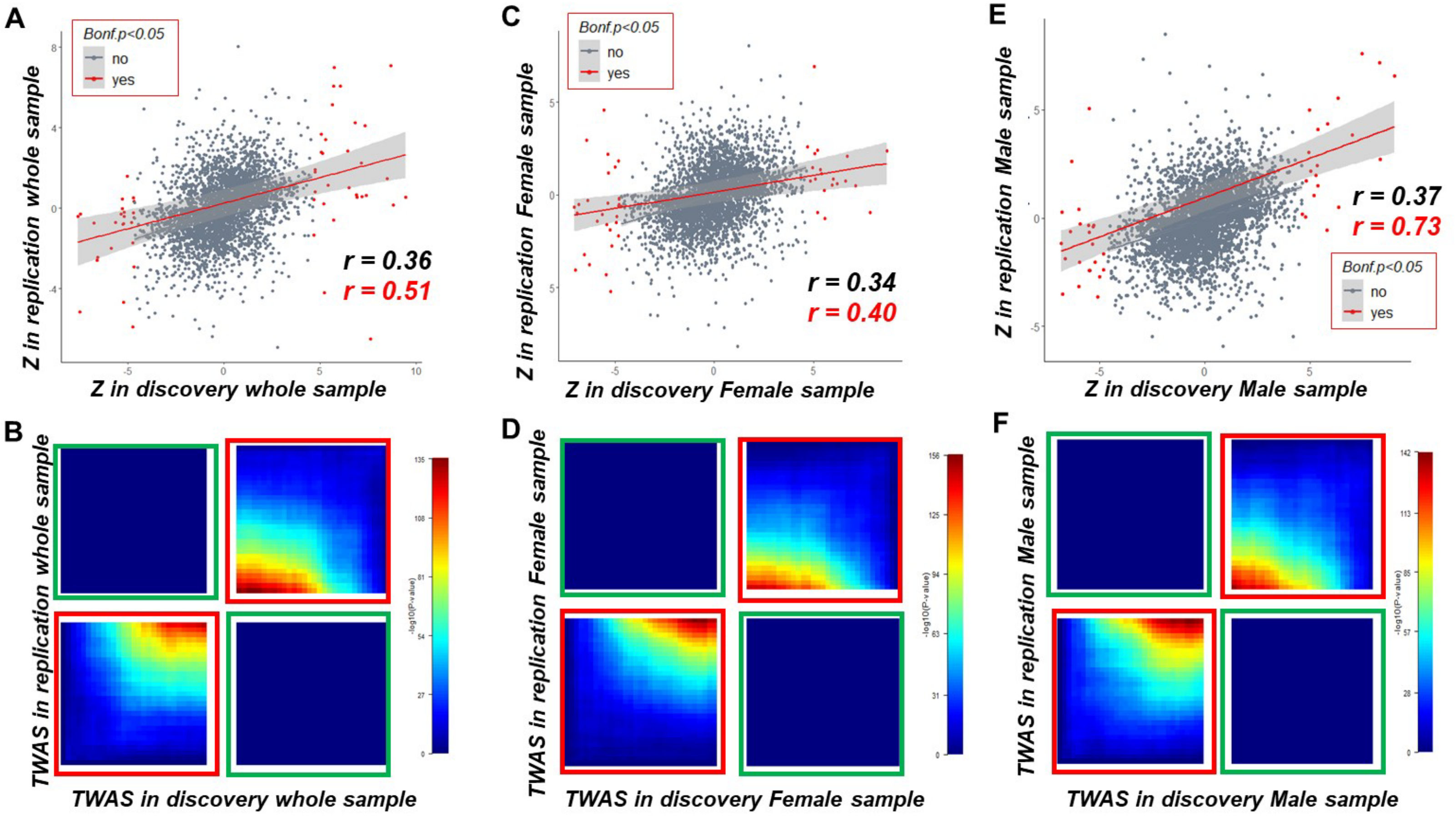

Supplementary Fig. 3: TWAS replication in placenta. Relationship between the Z-scores from the schizophrenia TWAS analyses, performed in the whole sample (**A,B**), in the female (**C,D**) and in the male sample (**E,F**). Z-scores are from the TWAS performed in the discovery placental dataset[117] (N=147: 73 females and 74 males) and in the replication placental dataset[116] (N=70: 35 females and 35 males). **A,C,E:** Scatterplots of the correlation between the Z scores from the TWAS analyses in the discovery dataset (x-axis) and in the replication dataset (y-axis), in the whole sample (**A**), and in placentae from female (**C**), and male (**E**) offspring. Genes TWAS-significant (Bonferroni-corrected p<0.05) in the discovery dataset are highlighted as red dots. Pearson correlation coefficients ("r") are reported for the total number of genes (black font) and for the TWAS-

significant genes in the discovery dataset (red font). Linear regression lines for the TWAS significant genes and the remaining genes are shown respectively in red and grey, with error bands indicating the 95% confidence region. **B,D,F:** RRHO2[118] heatmaps showing concordant (bottom-left and top-right quadrant in each panel, highlighted in a red frame) and discordant (top-left and bottom-right quadrant in each panel, highlighted in a green frame) TWAS association with schizophrenia in the discovery and in the replication dataset, in the whole sample (**B**), and in placentae from female (**D**) and male (**F**) offspring. Concordant and discordant associations are estimated using RRHO2, a threshold-free algorithm based on a rank-rank hypergeometric overlap approach[118]. Color bar represents negative logarithm of p-value of the overlap from the hypergeometric test.

Supplementary Information References:

1. Ferretti C, Bruni L, Dangles-Marie V, Pecking AP, Bellet D. Molecular circuits shared by placental and cancer cells, and their implications in the proliferative, invasive and migratory capacities of trophoblasts. *Hum Reprod Update* **13**, 121-141 (2007).

2. Kshitiz, *et al.* Evolution of placental invasion and cancer metastasis are causally linked. *Nat Ecol Evol* **3**, 1743-1753 (2019).

3. Naciri I, *et al.* Genetic screens reveal mechanisms for the transcriptional regulation of tissue-specific genes in normal cells and tumors. *Nucleic Acids Res* **47**, 3407-3421 (2019).

4. von Maltzahn J, Bentzinger CF, Rudnicki MA. Wnt7a-Fzd7 signalling directly activates the Akt/mTOR anabolic growth pathway in skeletal muscle. *Nat Cell Biol* **14**, 186-191 (2011).

5. Ray JP, *et al.* The Interleukin-2-mTORc1 Kinase Axis Defines the Signaling, Differentiation, and Metabolism of T Helper 1 and Follicular B Helper T Cells. *Immunity* **43**, 690-702 (2015).

6. Xu C, *et al.* TORC2/3-mediated DUSP1 upregulation is essential for human decidualization. *Reproduction* **161**, 573-580 (2021).

7. Chiba T, *et al.* Identification and characterization of an insulin receptor substrate 4-interacting protein in rat brain: implications for longevity. *Neurobiol Aging* **30**, 474-482 (2009).

8. Li J, *et al.* Intellectual disability-associated gene ftsj1 is responsible for 2'-O-methylation of specific tRNAs. *EMBO Rep* **21**, e50095 (2020).

9. She J, *et al.* The Alteration of m(6)A Modification at the Transcriptome-Wide Level in Human Villi During Spontaneous Abortion in the First Trimester. *Front Genet* **13**, 861853 (2022).

10. Xu XR, *et al.* Insight into hepatocellular carcinogenesis at transcriptome level by comparing gene expression profiles of hepatocellular carcinoma with those of corresponding noncancerous liver. *Proc Natl Acad Sci U S A* **98**, 15089-15094 (2001).

11. Vento-Tormo R, *et al.* Single-cell reconstruction of the early maternal-fetal interface in humans. *Nature* **563**, 347-353 (2018).

12. Young OM, *et al.* Toll-like receptor-mediated responses by placental Hofbauer cells (HBCs): a potential pro-inflammatory role for fetal M2 macrophages. *Am J Reprod Immunol* **73**, 22-35 (2015).

13. Li Y*, et al.* M860, a Monoclonal Antibody against Human Lactoferrin, Enhances Tumoricidal Activity of Low Dosage Lactoferrin via Granzyme B Induction. *Molecules* **24**, (2019).

14. Schwenk RW, Vogel H, Schurmann A. Genetic and epigenetic control of metabolic health. *Mol Metab* **2**, 337-347 (2013).

15. Mealer RG, Williams SE, Daly MJ, Scolnick EM, Cummings RD, Smoller JW. Glycobiology and schizophrenia: a biological hypothesis emerging from genomic research. *Molecular psychiatry* **25**, 3129-3139 (2020).

16. Deng Q, Liu X, Yang Z, Xie L. Expression of N-Acetylglucosaminyltransferase III Promotes Trophoblast Invasion and Migration in Early Human Placenta. *Reprod Sci* **26**, 1373-1381 (2019).

17. Howerton CL, Bale TL. Targeted placental deletion of OGT recapitulates the prenatal stress phenotype including hypothalamic mitochondrial dysfunction. *Proc Natl Acad Sci U S A* **111**, 9639-9644 (2014).

18. Sawade L*, et al.* Rab35-regulated lipid turnover by myotubularins represses mTORC1 activity and controls myelin growth. *Nat Commun* **11**, 2835 (2020).

19. Sun P, Bao A, Hua X, Cao J, Ding Y. RP5-1148A21.3 (lncRP5) exerts oncogenic function in human ovarian carcinoma. *Acta Biochim Biophys Sin (Shanghai)* **54**, 209-219 (2022).

20. Liu LZ*, et al.* Protein tyrosine phosphatase PTP4A1 promotes proliferation and epithelial-mesenchymal transition in intrahepatic cholangiocarcinoma via the PI3K/AKT pathway. *Oncotarget* **7**, 75210-75220 (2016).

21. Kajitani K, Yamaguchi H, Dan Y, Furuichi M, Kang D, Nakabeppu Y. MTH1, an oxidized purine nucleoside triphosphatase, suppresses the accumulation of oxidative damage of nucleic acids in the hippocampal microglia during kainate-induced excitotoxicity. *The Journal of neuroscience : the official journal of the Society for Neuroscience* **26**, 1688-1698 (2006).

22. Pudelko L*, et al.* Glioblastoma and glioblastoma stem cells are dependent on functional MTH1. *Oncotarget* **8**, 84671-84684 (2017).

23. Cham KL, Soga T, Parhar IS. RING Finger Protein 38 Is a Neuronal Protein in the Brain of Nile Tilapia, Oreochromis niloticus. *Front Neuroanat* **11**, 72 (2017).

24. Goeden N*, et al.* Maternal Inflammation Disrupts Fetal Neurodevelopment via Increased Placental Output of Serotonin to the Fetal Brain. *J Neurosci* **36**, 6041-6049 (2016).

25. Zhou J, Tang ZY, Sun XL. RNF38 inhibits osteosarcoma cell proliferation by binding to CRY1. *Biochem Cell Biol* **99**, 629-635 (2021).

26. Gong S*, et al.* The RNA landscape of the human placenta in health and disease. *Nat Commun* **12**, 2639 (2021).

27. Gu X*, et al.* DGKzeta Plays Crucial Roles in the Proliferation and Tumorigenicity of Human glioblastoma. *Int J Biol Sci* **15**, 1872-1881 (2019).

28. Huttlin EL*, et al.* Dual proteome-scale networks reveal cell-specific remodeling of the human interactome. *Cell* **184**, 3022-3040 e3028 (2021).

29. Scalise AA, Kakogiannos N, Zanardi F, Iannelli F, Giannotta M. The blood-brain and gut-vascular barriers: from the perspective of claudins. *Tissue Barriers* **9**, 1926190 (2021).

30. Ismail AA, Mahboob T, Samudi Raju C, Sekaran SD. Zika virus modulates blood-brain barrier of brain microvascular endothelial cells. *Trop Biomed* **36**, 888-897 (2019).

31. Ueda T*, et al.* Endothelial Cell-Selective Adhesion Molecule Contributes to the Development of Definitive Hematopoiesis in the Fetal Liver. *Stem Cell Reports* **13**, 992-1005 (2019).

32. Diener N*, et al.* Posttranslational modifications by ADAM10 shape myeloid antigen-presenting cell homeostasis in the splenic marginal zone. *Proc Natl Acad Sci U S A* **118**, (2021).

33. Adu-Gyamfi EA, Wang YX, Ding YB. The interplay between thyroid hormones and the placenta: a comprehensive reviewdagger. *Biol Reprod* **102**, 8-17 (2020).

34. Zhou Y*, et al.* Interactome analysis reveals ZNF804A, a schizophrenia risk gene, as a novel component of protein translational machinery critical for embryonic neurodevelopment. *Mol Psychiatry* **23**, 952-962 (2018).

35. Larsen ISB*, et al.* Discovery of an O-mannosylation pathway selectively serving cadherins and protocadherins. *Proc Natl Acad Sci U S A* **114**, 11163-11168 (2017).

36. Chen Y*, et al.* Methyltransferase-like 3 aggravates endoplasmic reticulum stress in preeclampsia by targeting TMBIM6 in YTHDF2-dependent manner. *Mol Med* **29**, 19 (2023).

37. Doycheva D, Xu N, Tang J, Zhang J. Viral-mediated gene delivery of TMBIM6 protects the neonatal brain via disruption of NPR-CYP complex coupled with upregulation of Nrf-2 post-HI. *J Neuroinflammation* **16**, 174 (2019).

38. Whitehead CL*, et al.* Identifying late-onset fetal growth restriction by measuring circulating placental RNA in the maternal blood at 28 weeks' gestation. *Am J Obstet Gynecol* **214**, 521 e521-521 e528 (2016).

39. Page NM, Dakour J, Morrish DW. Gene regulation of neurokinin B and its receptor NK3 in late pregnancy and pre-eclampsia. *Mol Hum Reprod* **12**, 427-433 (2006).

40. Juiz NA*, et al.* Alterations in Placental Gene Expression of Pregnant Women with Chronic Chagas Disease. *Am J Pathol* **188**, 1345-1353 (2018).

41. Schmitz MT*, et al.* The development and evolution of inhibitory neurons in primate cerebrum. *Nature* **603**, 871-877 (2022).

42. Boulet A*, et al.* The mammalian phosphate carrier SLC25A3 is a mitochondrial copper transporter required for cytochrome c oxidase biogenesis. *The Journal of biological chemistry* **293**, 1887-1896 (2018).

43. Fakhr Y, Brindley DN, Hemmings DG. Physiological and pathological functions of sphingolipids in pregnancy. *Cell Signal* **85**, 110041 (2021).

44. Oishi A, Karamitri A, Gerbier R, Lahuna O, Ahmad R, Jockers R. Orphan GPR61, GPR62 and GPR135 receptors and the melatonin MT(2) receptor reciprocally modulate their signaling functions. *Sci Rep* **7**, 8990 (2017).

45. Law J, Zhang G, Dragan M, Postovit LM, Bhattacharya M. Nodal signals via beta-arrestins and RalGTPases to regulate trophoblast invasion. *Cell Signal* **26**, 1935-1942 (2014).

46. Suter M*, et al.* Maternal tobacco use modestly alters correlated epigenome-wide placental DNA methylation and gene expression. *Epigenetics : official journal of the DNA Methylation Society* **6**, 1284-1294 (2011).

47. Wat MJ*, et al.* Recurrent microdeletions of 15q25.2 are associated with increased risk of congenital diaphragmatic hernia, cognitive deficits and possibly Diamond--Blackfan anaemia. *J Med Genet* **47**, 777-781 (2010).

48. Jiang X, Feng S, Chen Y, Feng Y, Deng H. Proteomic analysis of mTOR inhibition-mediated phosphorylation changes in ribosomal proteins and eukaryotic translation initiation factors. *Protein Cell* **7**, 533-537 (2016).

49. Evagelou SL, Bebenek O, Specker EJ, Uniacke J. DEAD Box Protein Family Member DDX28 Is a Negative Regulator of Hypoxia-Inducible Factor 2alpha- and Eukaryotic Initiation Factor 4E2-Directed Hypoxic Translation. *Mol Cell Biol* **40**, (2020).

50. Cao C*, et al.* Maternal Iron Deficiency Modulates Placental Transcriptome and Proteome in Mid-Gestation of Mouse Pregnancy. *J Nutr* **151**, 1073-1083 (2021).

51. Jain CV*, et al.* Regulation of HBEGF by Micro-RNA for Survival of Developing Human Trophoblast Cells. *PloS one* **11**, e0163913 (2016).

52. Nersisyan SA, Shkurnikov MY, Knyazev EN. Factors Involved in miRNA Processing Change Its Expression Level during Imitation of Hypoxia in BeWo b30 Cells. *Dokl Biochem Biophys* **493**, 205-207 (2020).

53. Cui Y*, et al.* Global miRNA dosage control of embryonic germ layer specification. *Nature* **593**, 602-606 (2021).

54. Paulson EE, Fishman EL, Schultz RM, Ross PJ. Embryonic microRNAs are essential for bovine preimplantation embryo development. *Proc Natl Acad Sci U S A* **119**, e2212942119 (2022).

55. Bortolin-Cavaille ML, Dance M, Weber M, Cavaille J. C19MC microRNAs are processed from introns of large Pol-II, non-protein-coding transcripts. *Nucleic Acids Res* **37**, 3464-3473 (2009).

56. Noguer-Dance M*, et al.* The primate-specific microRNA gene cluster (C19MC) is imprinted in the placenta. *Hum Mol Genet* **19**, 3566-3582 (2010).

57. Keverne EB. Genomic imprinting, action, and interaction of maternal and fetal genomes. *Proc Natl Acad Sci U S A* **112**, 6834-6840 (2015).

58. Petri R, Malmevik J, Fasching L, Akerblom M, Jakobsson J. miRNAs in brain development. *Exp Cell Res* **321**, 84-89 (2014).

59. Sun Y*, et al.* N(6)-methyladenosine-dependent pri-miR-17-92 maturation suppresses PTEN/TMEM127 and promotes sensitivity to everolimus in gastric cancer. *Cell Death Dis* **11**, 836 (2020).

60. Nallamshetty S, Chan SY, Loscalzo J. Hypoxia: a master regulator of microRNA biogenesis and activity. *Free Radic Biol Med* **64**, 20-30 (2013).

61. Gong WJ, Li R, Dai QQ, Yu P. METTL3 contributes to slow transit constipation by regulating miR-30b-5p/PIK3R2/Akt/mTOR signaling cascade through DGCR8. *J Gastroenterol Hepatol* **37**, 2229-2242 (2022).

62. Liu C*, et al.* Upregulation of CENPM facilitates lung adenocarcinoma progression via PI3K/AKT/mTOR signaling pathway. *Acta Biochim Biophys Sin (Shanghai)* **54**, 99-112 (2022).

63. Zheng C*, et al.* Upregulation of CENPM facilitates tumor metastasis via the mTOR/p70S6K signaling pathway in pancreatic cancer. *Oncol Rep* **44**, 1003-1012 (2020).

64. Leseva MN, *et al.* Differential gene expression and limited epigenetic dysregulation at the materno-fetal interface in preeclampsia. *Hum Mol Genet* **29**, 335-350 (2020).

65. Yin A, Chen Q, Zhong M, Jia B. MicroRNA-138 improves LPS-induced trophoblast dysfunction through targeting RELA and NF-kappaB signaling. *Cell Cycle* **20**, 508-521 (2021).

66. Fahlbusch FB, *et al.* Cullin 7 and Fbxw 8 expression in trophoblastic cells is regulated via oxygen tension: implications for intrauterine growth restriction? *J Matern Fetal Neonatal Med* **25**, 2209-2215 (2012).

67. Tsunematsu R, Nishiyama M, Kotoshiba S, Saiga T, Kamura T, Nakayama KI. Fbxw8 is essential for Cul1-Cul7 complex formation and for placental development. *Mol Cell Biol* **26**, 6157-6169 (2006).

68. Di Carlo P, Punzi G, Ursini G. Brain-derived neurotrophic factor and schizophrenia. *Psychiatr Genet* **29**, 200-210 (2019).

69. Liu L, Zhou X, Chen J, Li X. Potential of ATP5MG to Treat Metabolic Syndrome-Associated Cardiovascular Diseases. *Front Cardiovasc Med* **9**, 921778 (2022).

70. Wang X, *et al.* ATP5B promotes the metastasis and growth of gastric cancer by activating the FAK/AKT/MMP2 pathway. *FASEB J* **35**, e20649 (2021).

71. Roberts VH, Webster RP, Brockman DE, Pitzer BA, Myatt L. Post-Translational Modifications of the P2X(4) purinergic receptor subtype in the human placenta are altered in preeclampsia. *Placenta* **28**, 270-277 (2007).

72. Schmitt M, *et al.* Colon tumour cell death causes mTOR dependence by paracrine P2X4 stimulation. *Nature* **612**, 347-353 (2022).

73. Chatuphonprasert W, Jarukamjorn K, Ellinger I. Physiology and Pathophysiology of Steroid Biosynthesis, Transport and Metabolism in the Human Placenta. *Front Pharmacol* **9**, 1027 (2018).

74. McKenna MJ, *et al.* The endoplasmic reticulum P5A-ATPase is a transmembrane helix dislocase. *Science* **369**, (2020).

75. Wang Y, *et al.* Cell-type specific distribution and activation of type I IFN pathway molecules at the placental maternal-fetal interface in response to COVID-19 infection. *Front Endocrinol (Lausanne)* **13**, 951388 (2022).

76. Kwon JY, *et al.* Relevance of placental type I interferon beta regulation for pregnancy success. *Cell Mol Immunol* **15**, 1010-1026 (2018).

77. Zhu HL*, et al.* Melatonin protects against environmental stress-induced fetal growth restriction via suppressing ROS-mediated GCN2/ATF4/BNIP3-dependent mitophagy in placental trophoblasts. *Redox Biol* **40**, 101854 (2021).

78. Nuzzo AM*, et al.* Placental Adaptation to Early-Onset Hypoxic Pregnancy and Mitochondria-Targeted Antioxidant Therapy in a Rodent Model. *Am J Pathol* **188**, 2704-2716 (2018).

79. Ma Y, Chen Y, Lin C, Hu G. Biological functions and clinical significance of the newly identified long non-coding RNA RP1-85F18.6 in colorectal cancer. *Oncol Rep* **40**, 2648-2658 (2018).

80. Song J, Song W, Zhang L. LncRNA RP1-85F18.6 affects osteoblast cells by regulating the cell cycle. *Open Life Sci* **15**, 951-958 (2020).

81. Palma-Gudiel H, Eixarch E, Crispi F, Moran S, Zannas AS, Fananas L. Prenatal adverse environment is associated with epigenetic age deceleration at birth and hypomethylation at the hypoxia-responsive EP300 gene. *Clin Epigenetics* **11**, 73 (2019).

82. Jin M*, et al.* Regulator of G protein signaling 2 is inhibited by hypoxia-inducible factor-1alpha/E1A binding protein P300 complex upon hypoxia in human preeclampsia. *Int J Biochem Cell Biol* **147**, 106211 (2022).

83. Zhang Y*, et al.* Downregulation of decidual SP1 and P300 is associated with severe preeclampsia. *J Mol Endocrinol* **60**, 133-143 (2018).

84. Dowell J, Elser BA, Schroeder RE, Stevens HE. Cellular stress mechanisms of prenatal maternal stress: Heat shock factors and oxidative stress. *Neurosci Lett* **709**, 134368 (2019).

85. Kim SY*, et al.* Identifying Stabilin-1 and Stabilin-2 Double Knockouts in Reproduction and Placentation: A Descriptive Study. *Int J Mol Sci* **21**, (2020).

86. Palani S, Maksimow M, Miiluniemi M, Auvinen K, Jalkanen S, Salmi M. Stabilin-1/CLEVER-1, a type 2 macrophage marker, is an adhesion and scavenging molecule on human placental macrophages. *Eur J Immunol* **41**, 2052-2063 (2011).

87. Peng YR, Tran NM, Krishnaswamy A, Kostadinov D, Martersteck EM, Sanes JR. Satb1 Regulates Contactin 5 to Pattern Dendrites of a Mammalian Retinal Ganglion Cell. *Neuron* **95**, 869-883 e866 (2017).

88. Perez-Garcia V*, et al.* BAP1/ASXL complex modulation regulates epithelial-mesenchymal transition during trophoblast differentiation and invasion. *Elife* **10**, (2021).

89. Surguchov A. alpha-Synuclein and Mechanisms of Epigenetic Regulation. *Brain Sci* **13**, (2023).

90. Bononi A*, et al.* BAP1 is a novel regulator of HIF-1alpha. *Proc Natl Acad Sci U S A* **120**, e2217840120 (2023).

91. Khlghatyan J*, et al.* Fxr1 regulates sleep and synaptic homeostasis. *EMBO J* **39**, e103864 (2020).

92. Del'Guidice T*, et al.* FXR1P is a GSK3beta substrate regulating mood and emotion processing. *Proc Natl Acad Sci U S A* **112**, E4610-4619 (2015).

93. Khlghatyan J*, et al.* Mental Illnesses-Associated Fxr1 and Its Negative Regulator Gsk3beta Are Modulators of Anxiety and Glutamatergic Neurotransmission. *Front Mol Neurosci* **11**, 119 (2018).

94. Hong W, Chen JH, Ma HJ, Li L, Li XC. Fragile X-Related Protein 1 (FXR1) Promotes Trophoblast Migration at Early Pregnancy via Downregulation of GDF-15 Expression. *Reprod Sci* **29**, 110-121 (2022).

95. Okae H*, et al.* Re-investigation and RNA sequencing-based identification of genes with placenta-specific imprinted expression. *Hum Mol Genet* **21**, 548-558 (2012).

96. Chen J, Zhan Y, Xu J, Wang Y, Gao Q. EGR1 Overexpression Inhibits the Occurrence of Preeclampsia by Binding to MicroRNA-574 Promoter and Upregulating GAB1. *Reprod Sci* **28**, 1112-1121 (2021).

97. Zhou L*, et al.* Gab1 mediates PDGF signaling and is essential to oligodendrocyte differentiation and CNS myelination. *Elife* **9**, (2020).

98. Ryu HH*, et al.* Excitatory neuron-specific SHP2-ERK signaling network regulates synaptic plasticity and memory. *Sci Signal* **12**, (2019).

99. Bhaduri U, Merla G. Rise of TRIM8: A Molecule of Duality. *Mol Ther Nucleic Acids* **22**, 434-444 (2020).

100. Zhao W, Zhang X, Chen Y, Shao Y, Feng Y. Downregulation of TRIM8 protects neurons from oxygen-glucose deprivation/re-oxygenation-induced injury through reinforcement of the AMPK/Nrf2/ARE antioxidant signaling pathway. *Brain Res* **1728**, 146590 (2020).

101. Golob-Schwarzl N*, et al.* New liver cancer biomarkers: PI3K/AKT/mTOR pathway members and eukaryotic translation initiation factors. *Eur J Cancer* **83**, 56-70 (2017).

102. Kondoh K, Akahori H, Muto Y, Terada T. Identification of Key Genes and Pathways Associated with Preeclampsia by a WGCNA and an Evolutionary Approach. *Genes (Basel)* **13**, (2022).

103. Oguro-Ando A*, et al.* Increased CYFIP1 dosage alters cellular and dendritic morphology and dysregulates mTOR. *Mol Psychiatry* **20**, 1069-1078 (2015).

104. Yung HW*, et al.* Noncanonical mitochondrial unfolded protein response impairs placental oxidative phosphorylation in early-onset preeclampsia. *Proc Natl Acad Sci U S A* **116**, 18109-18118 (2019).

105. Gupta MB, Jansson T. Novel roles of mechanistic target of rapamycin signaling in regulating fetal growth. *Biol Reprod* **100**, 872-884 (2019).

106. Schrode N*, et al.* Synergistic effects of common schizophrenia risk variants. *Nat Genet* **51**, 1475-1485 (2019).

107. Zhou Z*, et al.* The proprotein convertase furin is required for trophoblast syncytialization. *Cell Death Dis* **4**, e593 (2013).

108. Zhou Z*, et al.* The proprotein convertase furin in human trophoblast: Possible role in promoting trophoblast cell migration and invasion. *Placenta* **30**, 929-938 (2009).

109. Gusev A*, et al.* Transcriptome-wide association study of schizophrenia and chromatin activity yields mechanistic disease insights. *Nat Genet* **50**, 538-548 (2018).

110. Du MR*, et al.* Cyclosporin A promotes growth and invasiveness in vitro of human first-trimester trophoblast cells via MAPK3/MAPK1-mediated AP1 and Ca2+/calcineurin/NFAT signaling pathways. *Biol Reprod* **78**, 1102-1110 (2008).

111. Diniz WJS*, et al.* Maternal Vitamin and Mineral Supplementation and Rate of Maternal Weight Gain Affects Placental Expression of Energy Metabolism and Transport-Related Genes. *Genes (Basel)* **12**, (2021).

112. Larkin JC, Sears SB, Sadovsky Y. The influence of ligand-activated LXR on primary human trophoblasts. *Placenta* **35**, 919-924 (2014).

113. Marseille-Tremblay C*, et al.* Impact of maternal circulating cholesterol and gestational diabetes mellitus on lipid metabolism in human term placenta. *Mol Reprod Dev* **75**, 1054-1062 (2008).

114. Ho WY*, et al.* TDP-43 mediates SREBF2-regulated gene expression required for oligodendrocyte myelination. *J Cell Biol* **220**, (2021).

115. Birolini G*, et al.* SREBP2 gene therapy targeting striatal astrocytes ameliorates Huntington's disease phenotypes. *Brain* **144**, 3175-3190 (2021).

116. Delahaye F*, et al.* Genetic variants influence on the placenta regulatory landscape. *PLoS Genet* **14**, e1007785 (2018).

117. Paquette AG, Lester BM, Koestler DC, Lesseur C, Armstrong DA, Marsit CJ. Placental FKBP5 genetic and epigenetic variation is associated with infant neurobehavioral outcomes in the RICHS cohort. *PLoS One* **9**, e104913 (2014).

118. Cahill KM, Huo Z, Tseng GC, Logan RW, Seney ML. Improved identification of concordant and discordant gene expression signatures using an updated rank-rank hypergeometric overlap approach. *Sci Rep* **8**, 9588 (2018).
