# Supplementary material for: Prioritization of potential causative genes for schizophrenia in placenta
Source: Nat Commun. 2023 May 15;14:2613. doi: 10.1038/s41467-023-38140-1 (PMC10185564; doi:10.1038/s41467-023-38140-1)
Supplement: Supplementary file 3 — Description of Additional Supplementary Files [file 41467_2023_38140_MOESM3_ESM.pdf]

Description of Additional Supplementary Files

Supplementary Data 1. Demographics of the placental samples. A) Total sample in placental TWAS for the analysis at gene and transcript level. Differences in the sample size are related to outlier detection (see Methods for details). **B**) Female sample in placental TWAS. **C**) Male sample in placental TWAS for the analysis at gene and transcript level. Differences in the sample size are related to outlier detection (see Methods for details).

Supplementary Data 2. Summary Statistics of placental TWAS for schizophrenia, at gene-level, in the whole sample.

Supplementary Data 3. Summary Statistics of placental TWAS for schizophrenia, in the whole sample, of the genes associated with schizophrenia with p<0.05, after Bonferroni-correction. Columns AA and AB indicate whether the TWAS genes are within the GWAS-significant loci at p<5e-08 (AA), and at p<1e-06 (AB). Red font highlights genes outside GWAS-significant loci (p<5e-08). For these genes, cell colors in columns Q, R, and S, indicate genes in the same loci.

Supplementary Data 4. Summary Statistics of placental TWAS for schizophrenia, at transcript-level, in the whole sample.

Supplementary Data 5. Summary Statistics of placental TWAS for schizophrenia, in the whole sample, of the transcripts associated with schizophrenia with p<0.05, after Bonferroni-correction. Columns AB and AC indicate whether the TWAS transcripts are within the GWAS-significant loci at p<5e-08 (AB), and at p<1e-06 (AC). Red font highlights transcripts outside GWAS-significant loci (p<5e-08). For these transcripts, cell colors in columns Q, R, and S, indicate transcripts in the same loci.

Supplementary Data 6. Summary Statistics of placental TWAS for schizophrenia, in the whole sample, of the genes and transcripts associated with schizophrenia with p<0.05, after Bonferroni-correction. A) Placental TWAS genes and transcripts associated with schizophrenia in the whole sample: Columns A-M report all the genes and transcripts. Column K specifies whether the association is at gene or transcript level. Column M reports the presence of transcripts associated with schizophrenia with opposite sign. **B) Unique genes:** Columns P-S report the unique placental genes associated with schizophrenia at gene and/or transcript level (for each gene the feature with the most significant association is selected).

Supplementary Data 7. Placental genes and transcripts with TWAS-significant association with schizophrenia, located outside GWAS-significant loci (p<5e-08).

Supplementary Data 8. Summary Statistics of placental SMR for schizophrenia, at gene-level, in the whole sample.

Supplementary Data 9. Summary Statistics of placental SMR for schizophrenia, at transcript-level, in the whole sample.

Supplementary Data 10. Placental unique genes with TWAS-significant association with schizophrenia at gene and/or transcript level, validated by SMR. Only TWAS genes with concordant (same sign) association with schizophrenia (p-SMR < 0.05, and with p-HEIDI > 0.01) are shown.

Supplementary Data 11: Pathway enrichment results. Reported are the results of the Ingenuity Pathway Analysis for the placental TWAS genes associated with schizophrenia in the whole sample. Statistics from right-tailed Fisher's Exact Test.

Supplementary Data 12: Function enrichment results. Reported are the results of the Ingenuity Global Functional Analysis for the placental TWAS genes associated with schizophrenia in the whole sample. Statistics from right-tailed Fisher's Exact Test.

Supplementary Data 13: Upstream Regulators enrichment results. Reported are the results of the Ingenuity Global Upstream Regulator Analysis for the placental TWAS genes associated with schizophrenia in the whole sample. Statistics from right-tailed Fisher's Exact Test.

Supplementary Data 14. Summary Statistics of placental TWAS for schizophrenia, at gene-level, in the female sample.

Supplementary Data 15. Summary Statistics of placental TWAS for schizophrenia, at transcript-level, in the female sample.

Supplementary Data 16. Summary Statistics of placental TWAS for schizophrenia, in the female sample, of the genes and transcripts associated with schizophrenia with $p<0.05$, after Bonferroni-correction. A) Placental TWAS genes and transcripts associated with schizophrenia in the female sample: Columns A-Q report all the genes and transcripts. Column Q specifies whether the association is at gene or transcript level. **B) Unique genes:** Columns U-Z report the unique placental genes associated with schizophrenia at gene and/or transcript level (for each gene the feature with the most significant association is selected) in the female sample. Column Z reports the presence of transcripts associated with schizophrenia wit opposite sign.

Supplementary Data 17. Summary Statistics of placental TWAS for schizophrenia, at gene-level, in the male sample.

Supplementary Data 18. Summary Statistics of placental TWAS for schizophrenia, at transcript-level, in the male sample.

Supplementary Data 19. Summary Statistics of placental TWAS for schizophrenia, in the male sample, of the genes and transcripts associated with schizophrenia with $p<0.05$, after Bonferroni-correction. A) Placental TWAS genes and transcripts associated with schizophrenia in the male sample: Columns A-Q report all the genes and transcripts. Column Q specifies whether the association is at gene or transcript level. **B) Unique genes:** Columns U-Z report the unique placental genes associated with schizophrenia at gene and/or transcript level (for each gene the feature with the most significant association is selected) in the male sample. Column Z reports the presence of transcripts associated with schizophrenia with opposite sign.

Supplementary Data 20. Placental unique genes with TWAS-significant association with schizophrenia at gene and/or transcript level, validated by SMR, in the female sample. Only TWAS genes with concordant (same sign) association with schizophrenia (p-SMR < 0.05, and with p-HEIDI > 0.01) are shown.

Supplementary Data 21. Placental unique genes with TWAS-significant association with schizophrenia at gene and/or transcript level, validated by SMR, in the male sample. Only TWAS genes with concordant (same sign) association with schizophrenia (p-SMR < 0.05, and with p-HEIDI > 0.01) are shown.

Supplementary Data 22. Overlap of TWAS-significant associations with schizophrenia in female and male placentae. A) TWAS-significant genes in both females and males, with Bonferroni corrected p <0.05: Columns A-I report the placental genes associated with schizophrenia with p<0.05 after Bonferroni correction. Column I shows the consistency of the sign of the TWAS-associations in female and male placentae. **B) TWAS-significant genes in both females and males - FDR corrected p <0.05:** Columns O-Z report the placental genes associated with schizophrenia with p<0.05 after FDR correction. Column Z indicates the consistency of the sign of the TWAS-associations in female and male placentae.

Supplementary Data 23. Coexpression of gene transcripts in female placentae. The table reports the output of the WGCNA analysis in female placentae, at transcript level. Column P (in red) indicates the module membership of each transcript. Columns K-U report measures of connectivity, that is, total connectivity (kTotal: connectivity of each gene based on its r-values to all other genes in the whole network), local connectivity (kWithin: connectivity of each gene within a single module based on its r-values to all other genes within the same module), and their differences (KOut, Kdiff).

Supplementary Data 24. Coexpression of gene transcripts in male placentae. The table reports the output of the WGCNA analysis in male placentae, at transcript level. Column P (in red) indicates the module membership of each transcript. Columns K-U report measures of connectivity, that is, total connectivity (kTotal: connectivity of each gene based on its r-values to all other genes in the whole network), local connectivity (kWithin: connectivity of each gene within a single module based on its r-values to all other genes within the same module), and their differences (KOut, Kdiff).

Supplementary Data 25. Transcript count overlap between WGCNA coexpression modules in male and female placentae. The table reports the number of overlapping transcripts between male (rows) and female (columns) modules generated by the WGCNA analysis in male and female placentae (see also Supplementary Figure 2).

Supplementary Data 26. Statistically significance of the overlap between WGCNA coexpression modules in male and female placentae. The table reports the uncorrected p-values of the Fisher exact test analyzing the overlap of transcripts between male (rows) and female (columns) modules generated by the WGCNA analysis in male and female placentae. Yellow cells indicate module-module overlaps significant (p<0.05) after Bonferroni-correction (see also Supplementary Figure 2).

Supplementary Data 27. Transcript count overlap between WGCNA coexpression modules in male and female placentae and placental TWAS schizophrenia genes. Shown are the number of placental TWAS schizophrenia transcripts detected in the whole sample (column D), in the female (E), and in the male sample (F), and also in each of the female-specific, male-specific, and overlapping modules (category in column A).

Supplementary Data 28: Pathway and upstream regulator's enrichment results of the placental coexpression modules containing schizophrenia TWAS transcripts. Reported are the results of the Ingenuity Pathway and Upstream Regulator Analysis for the coexpression modules containing the higher number (>10) of placental TWAS genes associated with schizophrenia in the whole sample, in female and male placentae. Statistics from right-tailed Fisher's Exact Test.

Supplementary Data 29. Demographics of the midgestational cerebral cortex samples.

Supplementary Data 30. Summary Statistics of prenatal cerebral cortex brain TWAS for schizophrenia, at gene-level, in the whole sample.

Supplementary Data 31. Summary Statistics of prenatal cerebral cortex TWAS for schizophrenia, at transcript-level, in the whole sample.

Supplementary Data 32. Summary Statistics of prenatal cerebral cortex TWAS for schizophrenia, in the whole sample, of the genes and transcripts associated with schizophrenia with p<0.05, after Bonferroni-correction. A) Prenatal cortical brain TWAS genes and transcripts associated with schizophrenia in the whole sample: Columns A-Q report all the genes and transcripts with TWAS-significant association with schizophrenia. Column Q specifies whether the association is at gene or transcript level. **B) Unique genes:** Columns V-AD report the unique prenatal cerebral cortex genes associated with schizophrenia at gene and/or transcript level (for each gene the feature with the most significant association is selected). Column AD indicate the presence of transcripts associate with schizophrenia with opposite sign.

Supplementary Data 33. Summary Statistics of prenatal cerebral cortex TWAS for schizophrenia, at gene-level, in the female sample.

Supplementary Data 34. Summary Statistics of prenatal cerebral cortex TWAS for schizophrenia, at transcript-level, in the female sample.

Supplementary Data 35. Unique genes with TWAS-significant association with schizophrenia, at gene and/or transcript level, in prenatal cerebral cortex, in the female sample: Columns A-I report the unique prenatal cortical brain genes associated with schizophrenia at gene and/or transcript level (for each gene the feature with the most significant association is selected). Column I indicate the presence of transcripts associate with schizophrenia with opposite sign.

Supplementary Data 36. Summary Statistics of prenatal cerebral cortex TWAS for schizophrenia, at gene-level, in the male sample.

Supplementary Data 37. Summary Statistics of prenatal cerebral cortex TWAS for schizophrenia, at transcript-level, in the male sample.

Supplementary Data 38. Unique genes with TWAS-significant association with schizophrenia, at gene and/or transcript level, in prenatal cerebral cortex, in the male sample: Columns A-I report the unique prenatal cortical brain genes associated with schizophrenia at gene and/or transcript level (for each gene the feature with the most significant association is selected). Column I indicate the presence of transcripts associate with schizophrenia with opposite sign.

Supplementary Data 39. Genes with TWAS-significant association with schizophrenia, at gene and/or transcript level, in prenatal cerebral cortex, in the whole sample, in female, and in male. A) The table reports all the TWAS significant genes, with p<0.05 after Bonferroni-correction. Column I indicates the presence of transcripts associated with schizophrenia with opposite sign. **B)** The table reports the TWAS significant genes in male and female prenatal cortical brains, with p<0.05 after Bonferroni-correction. **C)** The table reports the unique TWAS genes associated with schizophrenia in the whole sample, in male and females prenatal cortical brains, at gene and/or transcript level, with p<0.05 after Bonferroni-correction.

Supplementary Data 40. Larger set of genes with TWAS association with schizophrenia, at gene and/or transcript level, in prenatal brain, in the whole sample, in female, and in male. A) The table reports all the TWAS significant genes, with uncorrected p<0.05. **B)** The table reports the TWAS significant genes in male and female prenatal cortical brains, with p<0.05 after FDR-correction.

Supplementary Data 41. Placental-specific TWAS genes associated with schizophrenia. A) The table reports all the TWAS genes associated with schizophrenia in placenta with p<0.05 after Bonferroni-correction, which do not show association with schizophrenia in prenatal brain (FDR>0.01). Column F reports whether the gene has been associated with schizophrenia by SMR analysis in fetal and adult brain and in blood (PMID: 35396580). **B) Pathway enrichment results of placental-specific TWAS genes:** Reported are the results of the Ingenuity Pathway Analysis for the placental-specific TWAS genes associated with schizophrenia. Statistics from right-tailed Fisher's Exact Test. **C) Upstream Regulators enrichment results of placental-specific TWAS genes:** Reported are the results of the Ingenuity Global Upstream Regulator Analysis for the placental-specific TWAS genes associated with schizophrenia. Statistics from right-tailed Fisher's Exact Test.

Supplementary Data 42. Prenatal cortex-specific TWAS genes associated with schizophrenia. The table reports all the TWAS genes associated with schizophrenia in midgestational cortical brain with p<0.05 after Bonferroni-correction, which do not show association with schizophrenia in placenta.

Supplementary Data 43. Placenta&brain-pleiotropic TWAS genes associated with schizophrenia. A) The table reports all the TWAS genes associated with schizophrenia in placenta and in prenatal cerebral cortex with p<0.05 after Bonferroni-correction. Columns H indicates whether the association has opposite sign in brain and placenta. **B) Pathway enrichment results of placenta&brain-pleiotropic TWAS genes:** Reported are the results of the Ingenuity Pathway Analysis for the placenta&brain-pleiotropic TWAS genes associated with schizophrenia, with predicted z-scores in placenta (P) and in fetal brain (Q). Statistics from right-tailed Fisher's Exact Test. **C) Upstream Regulators enrichment results of placenta&brain-pleiotropic TWAS genes:** Reported are the results of the Ingenuity Global Upstream Regulator Analysis for the placenta&brain-pleiotropic TWAS genes associated with schizophrenia, with predicted activation state and Z-scores in placenta (AB, AD) and fetal brain (AC, AE). Statistics from right-tailed Fisher's Exact Test.

Supplementary Data 44. Placental TWAS-significant associations with autism spectrum disorder (Bonferroni-corrected p<0.05). Columns S and T report gene variant-level colocalization probability (GRCP) and gene locus-level colocalization probability (GLCP), from the colocalization analysis.

Supplementary Data 45. Placental TWAS-significant associations with ADHD (Bonferroni-corrected p<0.05). A) Placental TWAS genes and transcripts associated with ADHD in the whole sample: Columns A-O report all the genes and transcripts. **B) Unique genes:** Columns S-X report the unique placental genes associated with ADHD at gene and/or transcript level (for each gene the feature with the most significant association is selected). Column X indicates the presence of transcripts associated with ADHD with opposite sign. Columns Y-AB report gene variant-level colocalization probability (GRCP) and gene locus-level colocalization probability (GLCP), from the colocalization analyses at gene-level (Y, Z) and transcript level (AA, AB).

Supplementary Data 46. Placental TWAS-significant associations with major depression (Bonferroni-corrected p<0.05). A) Placental TWAS genes and transcripts associated with depression in the whole sample: Columns A-O report all the genes and transcripts. **B) Unique genes:** Columns S-X report the unique placental genes associated with depression at gene and/or transcript level (for each gene the feature with the most significant association is selected). Column X indicates the presence of transcripts associated with depression with opposite sign. Columns Y-AB report gene variant-level colocalization probability (GRCP) and gene locus-level colocalization probability (GLCP), from the colocalization analyses at gene-level (Y, Z) and transcript level (AA, AB).

Supplementary Data 47. Placental TWAS-significant associations with bipolar disorder (Bonferroni-corrected p<0.05). A) Placental TWAS genes and transcripts associated with bipolar disorder in the whole sample: Columns A-O report all the genes and transcripts. **B) Unique genes:** Columns S-X report the unique placental genes associated with bipolar disorder at gene and/or transcript level (for each gene the feature with the most significant association is selected). Column X indicates the presence of transcripts associated with bipolar disorder with opposite sign. Columns Y-AB report gene variant-level colocalization probability (GRCP) and gene locus-level colocalization probability (GLCP), from the colocalization analyses at gene-level (Y, Z) and transcript level (AA, AB).

Supplementary Data 48. Placental TWAS-significant associations with birthweight (Bonferroni-corrected p<0.05). A) Placental TWAS genes and transcripts associated with birthweight in the whole sample: Columns A-O report all the genes and transcripts. **B) Unique genes:** Columns S-X report the unique placental genes associated with birthweight at gene and/or transcript level (for each gene the feature with the most significant association is selected). Column X indicates the presence of transcripts associated with birthweight with opposite sign. Columns Y-AB report gene variant-level colocalization probability (GRCP) and gene locus-level colocalization probability (GLCP), from the colocalization analyses at gene-level (Y, Z) and transcript level (AA, AB).

Supplementary Data 49. Placental TWAS-significant associations with height (Bonferroni-corrected p<0.05). A) Placental TWAS genes and transcripts associated with height in the whole sample: Columns A-O report all the genes and transcripts. **B) Unique genes:** Columns S-X report the unique placental genes associated with height at gene and/or transcript level (for each gene the feature with the most significant association is selected). Column X indicates the presence of transcripts associated with height with opposite sign. Columns Y-AB report gene variant-level colocalization probability (GRCP) and gene locus-level colocalization probability (GLCP), from the colocalization analyses at gene-level (Y, Z) and transcript level (AA, AB).

Supplementary Data 50. Placental TWAS-significant associations with BMI (Bonferroni-corrected p<0.05). A) Placental TWAS genes and transcripts associated with BMI in the whole sample: Columns A-O report all the genes and transcripts. **B) Unique genes:** Columns S-X report the unique placental genes associated with BMI at gene and/or transcript level (for each gene the feature with the most significant association is selected). Column X indicates the presence of transcripts associated with BMI with opposite sign. Columns Y-AB report gene variant-level colocalization probability (GRCP) and gene locus-level colocalization probability (GLCP), from the colocalization analyses at gene-level (Y, Z) and transcript level (AA, AB).

Supplementary Data 51. Placental TWAS-significant associations with type 2 diabetes (Bonferroni-corrected p<0.05). A) Placental TWAS genes and transcripts associated with diabetes in the whole sample: Columns A-O report all the genes and transcripts. **B) Unique genes:** Columns S-X report the unique placental genes associated with diabetes at gene and/or transcript level (for each gene the feature with the most significant association is selected). Column X indicates the presence of transcripts associated with diabetes with opposite sign. Columns Y-AB report gene variant-level colocalization probability (GRCP) and gene locus-level colocalization probability (GLCP), from the colocalization analyses at gene-level (Y, Z) and transcript level (AA, AB).

Supplementary Data 52. Placental TWAS-significant associations with insomnia at gene and transcript level (uncorrected p<0.001).

Supplementary Data 53. Placental TWAS-significant associations with IQ (Bonferroni-corrected p<0.05). A) Placental TWAS genes and transcripts associated with IQ in the whole sample: Columns

A-O report all the genes and transcripts. **B) Unique genes:** Columns S-X report the unique placental genes associated with IQ at gene and/or transcript level (for each gene the feature with the most significant association is selected). Column X indicates the presence of transcripts associated with IQ with opposite sign. Columns Y-AB report gene variant-level colocalization probability (GRCP) and gene locus-level colocalization probability (GLCP), from the colocalization analyses at gene-level (Y, Z) and transcript level (AA, AB).

Supplementary Data 54. Placenta&Schizophrenia prioritized risk genes. List of 502 genes whose expression in placenta at gene and/or transcript level is predicted to be associated with schizophrenia, in the whole sample, and/or in male, and/or in female placentae (sample with the most significant association in column F with statistics in columns D,E; statistics in all samples in columns AA-AL). Column L reports whether the gene has TWAS-association with schizophrenia in fetal brain with FDR<0.01. Columns M-O reports whether the gene has been prioritized by the most recent schizophrenia GWAS (PMID: 35396580) based on fine mapping (M), SMR in adult and fetal brain and blood (N), or being annotated as a rare-variant priority gene (O). Columns P-Y indicate placental TWAS associations with other disorders and traits. Columns AM-BC summarize the results of the colocalization (COLOC) analysis: GRCP, that is, gene variant-level colocalization probability, in the analysis at gene level in the whole sample (AR), in the female (AV) and in the male (AZ) samples; GLCP, that is, gene locus-level colocalization probability, in the analysis at gene level in the whole sample (AS), in the female (AW) and in the male (BA) samples; GRCP in the analysis at transcript level in the whole sample (AT), in the female (AX) and in the male (BB) samples; GLCP in the analysis at transcript level in the whole sample (AU), in the female (AY) and in the male (BC) samples; maximum value of GRCP is reported in column AO, maximum value of GLCP is reported in column AP, maximum value of GRCP or GLCP is reported in column AQ; columns AM and AN report whether a gene has GRCP or GLCP > 0.5 (AM) and 0.1 (AN). Column J highlights whether the placental schizophrenia genes are prioritized based on absence of association in brain and absence of relevant association with other disorders and traits in placenta. Validation of association with schizophrenia with SMR is indicated in column K. The 139 prioritized genes validated with SMR are indicated in column I. In column I and J, we also report whether a prioritized gene has GRCP or GLCP higher than 0.5 or 0.1 (top-prioritized genes in column I). Columns Z indicates whether the placental TWAS gene has been detected in maternal blood as a cell-free RNA biomarker of pregnancy outcomes (PMID: 35140405). Recent changes of gene annotations are indicated in column C with Entrez Gene Name and other gene information. Gene synonyms, location and types are reported in columns BD, BE, and BF, respectively. See also Supplementary Fig. 1 for workflow on the prioritization of placenta&schizophrenia- risk genes, Supplementary Data 56-61 for detailed results from colocalization analysis, and Supplementary Data 55 and Supplementary Note 1 for biological information about selected genes.

Supplementary Data 55. Master regulators, diseases and functions, associated with the top-prioritized placenta- schizophrenia-specific risk genes. **A) Causal Network Analysis.** Reported in columns A-J are the results of the causal network analysis, in IPA, to identify master regulators of the 33 top-prioritized placenta- schizophrenia-specific risk, which show TWAS-significant association with schizophrenia in placenta, and not in fetal brain, are validated by SMR and colocalization analyses, and did not show any significant relevant association with any of the other disorders and traits analyzed. P-value of overlap is obtained by right-tailed Fisher's Exact Test; Network bias-corrected p-value is calculated by explicit permutation sampling to account for the presence of network hub genes in the list of top-prioritized placenta- schizophrenia-specific risk genes (PMID: 24336805). **B) Isoprofiler analysis.** Reported in columns P-AC are the results of the Isoprofiler analysis in IPA to identify functions and diseases terms associated with the top-prioritized placenta- schizophrenia-specific risk (please note that

not all the 33 top-prioritized genes have known annotations). See also Supplementary Note 1 for biological information about selected genes.

Supplementary Data 56. Colocalization results from the gene-level analysis in the whole sample of placentae (N=146). Reported is the output from fastENLOC.

Supplementary Data 57. Colocalization results from the gene-level analysis in the sample of placentae from female offspring (N=73). Reported is the output from fastENLOC.

Supplementary Data 58. Colocalization results from the gene-level analysis in the sample of placentae from male offspring (N=73). Reported is the output from fastENLOC.

Supplementary Data 59. Colocalization results from the transcript-level analysis in the whole sample of placentae (N=147). Reported is the output from fastENLOC.

Supplementary Data 60. Colocalization results from the transcript-level analysis in the sample of placentae from female offspring (N=73). Reported is the output from fastENLOC.

Supplementary Data 61. Colocalization results from the transcript-level analysis in the sample of placentae from male offspring (N=74). Reported is the output from fastENLOC.

For Supplementary Data 56-61:

- Spreadsheet **1** contains the Enrichment analysis results ("prefix.enloc.enrich.rst"): estimated enrichment parameters and standard errors.
- Spreadsheet **2** contains Gene/Transcript-level colocalization result ("prefix.enloc.gene.out"): Gene/Transcript-level colocalization output with the following format:
  - column A: Gene/Transcript name
  - column B: Gene/Transcript variant-level colocalization probability (GRCP)
  - column C: Gene/Transcript locus-level colocalization probability (GLCP)
- Spreadsheet **3** contains Signal-level colocalization result ("prefix.enloc.sig.out") with the following format:
  - column A: signal cluster name (from eQTL analysis)
  - column B: number of member SNPs
  - column C: cluster PIP of eQTLs
  - column D: cluster PIP of GWAS hits (*without* eQTL prior)
  - column E: cluster PIP of GWAS hits (*with* eQTL prior)
  - column F: regional colocalization probability (RCP)
  - column G: locus-level colocalization probability (LCP)
- Spreadsheet **4** contains SNP-level colocalization result ("prefix.enloc.snp.out"): SNP-level colocalization output with the following format"
  - column A: signal cluster name
  - column B: SNP name
  - column C: SNP-level PIP of eQTLs
  - column D: SNP-level PIP of GWAS (*without* eQTL prior)
  - column E: SNP-level PIP of GWAS (*with* eQTL prior)
  - column F: SNP-level colocalization probability

Supplementary Data 62. Demographics of the placenta replication sample. A) Total sample in placental TWAS for the replication analysis. **B**) Female sample in placental replication TWAS. **C**) Male sample in placental replication TWAS.

Supplementary Data 63. Summary Statistics of placental replication TWAS for schizophrenia, in the whole sample, and comparison with the discovery sample. Columns K-T (top row in blue font) report summary statistics of the replication TWAS; for comparison, summary statistics of the same gene in the discovery sample are reported in columns E-J (top row in red font).

Supplementary Data 64. Summary Statistics of placental replication TWAS for schizophrenia, in the female sample, and comparison with the female discovery sample. Columns K-T (top row in blue font) report summary statistics of the replication TWAS; for comparison, summary statistics of the same gene in the discovery sample are reported in columns E-J (top row in red font).

Supplementary Data 65. Summary Statistics of placental replication TWAS for schizophrenia, in the male sample, and comparison with the male discovery sample. Columns K-T (top row in blue font) report summary statistics of the replication TWAS; for comparison, summary statistics of the same gene in the discovery sample are reported in columns E-J (top row in red font).

Supplementary Data 66. Summary Statistics of placental TWAS for schizophrenia, at gene-level, in the whole sample, in the MHC locus.

Supplementary Data 67. Summary Statistics of placental TWAS for schizophrenia, at gene-level, in the female sample, in the MHC locus.

Supplementary Data 68. Summary Statistics of placental TWAS for schizophrenia, at gene-level, in the male sample, in the MHC locus.

Supplementary Data 69. Summary Statistics of placental TWAS for schizophrenia, at transcript-level, in the whole sample, in the MHC locus.

Supplementary Data 70. Summary Statistics of placental TWAS for schizophrenia, at transcript-level, in the female sample, in the MHC locus.

Supplementary Data 71. Summary Statistics of placental TWAS for schizophrenia, at transcript-level, in the male sample, in the MHC locus.
