# Supplementary material for: Prioritization of potential causative genes for schizophrenia in placenta
Source: Nat Commun. 2023 May 15;14:2613. doi: 10.1038/s41467-023-38140-1 (PMC10185564; doi:10.1038/s41467-023-38140-1)
Supplement: Supplementary file 16 — Reporting Summary [file 41467_2023_38140_MOESM16_ESM.pdf]

nature portfolio

Corresponding author(s): Daniel R. Weinberger; Gianluca Ursini

Last updated by author(s): Mar 21, 2023

Reporting Summary

Nature Portfolio wishes to improve the reproducibility of the work that we publish. This form provides structure for consistency and transparency in reporting. For further information on Nature Portfolio policies, see our Editorial Policies and the Editorial Policy Checklist.

Statistics

For all statistical analyses, confirm that the following items are present in the figure legend, table legend, main text, or Methods section.

n/a	Confirmed	
☐	☒	The exact sample size (*n*) for each experimental group/condition, given as a discrete number and unit of measurement
☐	☒	A statement on whether measurements were taken from distinct samples or whether the same sample was measured repeatedly
☐	☒	The statistical test(s) used AND whether they are one- or two-sided<br>*Only common tests should be described solely by name; describe more complex techniques in the Methods section.*
☐	☒	A description of all covariates tested
☐	☒	A description of any assumptions or corrections, such as tests of normality and adjustment for multiple comparisons
☐	☒	A full description of the statistical parameters including central tendency (e.g. means) or other basic estimates (e.g. regression coefficient) AND variation (e.g. standard deviation) or associated estimates of uncertainty (e.g. confidence intervals)
☐	☒	For null hypothesis testing, the test statistic (e.g. *F*, *t*, *r*) with confidence intervals, effect sizes, degrees of freedom and *P* value noted<br>*Give P values as exact values whenever suitable.*
☒	☐	For Bayesian analysis, information on the choice of priors and Markov chain Monte Carlo settings
☒	☐	For hierarchical and complex designs, identification of the appropriate level for tests and full reporting of outcomes
☐	☒	Estimates of effect sizes (e.g. Cohen's *d*, Pearson's *r*), indicating how they were calculated

Our web collection on statistics for biologists contains articles on many of the points above.

Software and code

Policy information about availability of computer code

Data collection

RNA sequencing data were downloaded using the SRA toolkit (version 3.0.0).

Data analysis

For RNA sequencing data processing, gene- and transcript-level quantification was obtained using the SPEAQeasy pipeline described in https://github.com/LieberInstitute/SPEAQeasy (downloaded in 2021; ref.91). We used PLINK (version 1.9; www.cog-genomics.org/plink/1.9/ ; ref.96,97) for genotype data processing and quality check. We performed principle components analysis on independent SNPs with smartpca in EIGENSOFT (version 8.0.0; ref.98).
TWAS was performed using fusion TWAS (downloaded from github in 2021), as described at http://gusevlab.org/projects/fusion/ (ref.22). We used GCTA (version 1.94.0, ref.100) to estimate cis SNP heritability for each gene. We used a threshold-free algorithm, RRHO2 (ref.60; downloaded from github in 2022), to detect pattern of concordant and discordant TWAS association of schizophrenia with the other disorders and traits, and to compare TWAS results in the discovery and replication sample. SMR and HEIDI were performed using the SMR software tool (version 1.3.1, https://yanglab.westlake.edu.cn/software/smr/#Overview; ref.25). WGCNA (ref.55,95) was performed using the WGCNA package (version 1.69-81) in R (version 4.0). Following recommendations from the LDSC resource website (https://alkesgroup.broadinstitute.org/LDSCORE/ ; ref.111), S-LDSC was run for each list of variants with the baseline LD model v2.2. To perform colocalization analysis, we first implemented eQTL fine mapping by estimating priors from the MatrixQTL nominal results with the TORUS method (ref.112). Following estimation of priors, we implemented DAP-G (ref.67,113) to generate posterior inclusion probabilities that provide an estimate of the probability of a variant being causal for downstream colocalization with fastENLOC (version 2.0; ref.68).
Other statistical analyses were performed in the R environment (version 4.0 and 4.1.3); the package limma (version 3.40.6 in Bioconductor 3.9; ref.114) was used for 'geneSetTest'. Bioturing BBrowser (version 2.10.40; ref.116; www.bioturing.com) was used to explore single cell data and to generate Fig. 5. Low expressed genes in the placental and fetal brain RNA sequencing datasets and also in SARS-CoV-2 dataset were identified using the expression_cutoff function in the jaffelab R package (version 0.99.31; ref.92). Pathway, functional, and upstream regulator analyses were performed using the QIAGEN's Ingenuity Pathway Analysis software (IPA, QIAGEN, Redwood City, CA, USA; http://

www.qiagen.com/ingenuity; all the analyses performed with the April 2022 release of IPA, except the causal network and Isoprofiler of Supplementary Data 55, which were performed with the December 2022 release version; results were consistent when repeating all the analyses with the December 2022 release).

For manuscripts utilizing custom algorithms or software that are central to the research but not yet described in published literature, software must be made available to editors and reviewers. We strongly encourage code deposition in a community repository (e.g. GitHub). See the Nature Portfolio guidelines for submitting code & software for further information.

Data

Policy information about availability of data

All manuscripts must include a data availability statement. This statement should provide the following information, where applicable:

- Accession codes, unique identifiers, or web links for publicly available datasets
- A description of any restrictions on data availability
- For clinical datasets or third party data, please ensure that the statement adheres to our policy

All the data analyzed in this manuscript are publicly available and can be obtained via authorized access from the dbGaP database. Placenta RNA seq and genotype data are available on the NCBI SRA public database under accession code SRP095910 and on the dbGaP database under accession code phs001586.v1.p1 (discovery sample), and under accession code phs001717.v1.p1 (replication sample). Midgestational prenatal cortical brain RNAseq data are available on the dbGaP database under accession code phs001900.v1.p1.

Human research participants

Policy information about studies involving human research participants and Sex and Gender in Research.

Reporting on sex and gender	We performed our TWAS analyses in whole sample, and stratifying by sex. The placental discovery dataset included 73 placentae from female offspring, and 74 placentae from male offspring. The placental replication dataset included 35 placentae from female offspring, and 35 placentae from male offspring. The midgestational prenatal cortical brain dataset included 67 female samples, and 99 male samples.
Population characteristics	Gestational age in the placental discovery sample was 39.07 weeks +/- 0.97. Gestational age in the placental discovery sample was 39.39 weeks +/- 1.27. Gestational age in the midgestational prenatal cortical brain sample was 17.15 weeks +/- 1.71.
Recruitment	Placenta tissues of the discovery samples were collected as part of the Rhode Island Child Health Study (RICHS, ref.89). This population consists of singleton, term infants born without serious pregnancies complications or congenital or chromosomal abnormalities.<br>For the placenta replication study we used data from the Eunice Kennedy Shriver National Institute of Child Health and Human Development (NICHD) Fetal Growth Study (ref.74), which was a prospective, multicentered, observational study (sample size = 70 for this study). Healthy, non-obese, low risk pregnant women across four race/ethnicity groups, who conceived spontaneously and had no obvious risk factors for fetal growth restriction or overgrowth were eligible for inclusion in the study. All women were between 18 and 40 years old with BMI between 19.0 to 29.9kg/m2 with no confirmed or suspected fetal congenital structural or chromosomal anomalies. Multiple exclusions were identified to assure a low risk population and included but was not limited to, cigarette smoking in the past six months, use of illicit drugs in the past year, consumption of at least 1 alcoholic drink per day, chronic hypertension, diabetes mellitus, HIV or AIDS, and history of gestational diabetes in a prior pregnancy. To assure correct dating, all pregnancies had first trimester ultrasound screening consistent with gestational age. Placentas were collected from pregnancies with a predicted birthweight by ultrasound between the 10th and the 90th percentile (ref.74).<br>For the study in midgestational prenatal cortical brain, we used RNAseq data from prenatal tissue, which was obtained from the UCLA Gene and Cell Therapy core according to IRB guidelines from 233 donors (sample size =166 for this study) following voluntary termination of pregnancy (ref.33).
Ethics oversight	We used publicly available data, which were collected under protocols IRB-approved from each Institution.<br>- Placenta discovery dataset: the placenta data of the discovery dataset were part of the RICHS study (ref.89): the protocol of this study was reviewed and approved by the Institutional Review Boards at Women and Infants Hospital of Rhode Island and Emory University.<br>- Placenta replication dataset: the placenta data of the replication dataset were generated in the context of a study (ref.74) that has been approved by the Columbia University IRB Exp (protocol number is IRB-AAAD9014).<br>- Midgestational prenatal cortical brain dataset: these data were generated from tissue obtained from the UCLA Gene and Cell Therapy core according to IRB guidelines; this study (ref.33) was performed under the auspices of the UCLA Office of Human Research Protection, which determined that it was exempt because samples are anonymous pathological specimens.

Note that full information on the approval of the study protocol must also be provided in the manuscript.

Field-specific reporting

Please select the one below that is the best fit for your research. If you are not sure, read the appropriate sections before making your selection.

☒ Life sciences ☐ Behavioural & social sciences ☐ Ecological, evolutionary & environmental sciences

For a reference copy of the document with all sections, see nature.com/documents/nr-reporting-summary-flat.pdf

Life sciences study design

All studies must disclose on these points even when the disclosure is negative.

Sample size	Sample size was not predetermined. We used all the placenta and prenatal cortical brain RNAseq data available from the downloaded datasets, which survived QC.
Data exclusions	We dropped one placental sample from our analyses in the discovery sample, because it had low overall alignment rate with high chrM alignment rate. We also computed samples' pairwise correlations (Pearsons's r) to define relative Euclidean distance among subjects. Then, we excluded outliers when the deviation from the average distance is ≥ 3 standard deviations.
Replication	Replication was performed in a dataset with a smaller sample size, in the whole sample (N=70), and also in female (N=35) and male (N=35) placentae. We analyzed the correlation between Z-scores in the discovery and replication samples, and we also used the RRHO2 algorithm (Fig.S3, ref60) to verify the presence of pattern of concordance between the two studies. We also performed a Chi-square test with Yates correction to verify whether the number of TWAS -significant genes in the discovery sample were replicated ($p<0.05$) with the same directionality in the replication sample. Attempt of replication was successful.
Randomization	Randomization is not applicable to this study, because there are not experimental groups. The datasets analyzed are from observational studies, which did not include randomized experiments.
Blinding	Genotyping and RNA sequencing were performed by investigators of previous studies, who were therefore blinded to the future use of the data. The analyses described in this study were performed using analytical pipelines where the researcher is necessarily blinded to the gene expression and genotype data of each individual.

Reporting for specific materials, systems and methods

We require information from authors about some types of materials, experimental systems and methods used in many studies. Here, indicate whether each material, system or method listed is relevant to your study. If you are not sure if a list item applies to your research, read the appropriate section before selecting a response.

Materials & experimental systems

n/a	Involved in the study	
☒	☐	Antibodies
☒	☐	Eukaryotic cell lines
☒	☐	Palaeontology and archaeology
☒	☐	Animals and other organisms
☒	☐	Clinical data
☒	☐	Dual use research of concern

Methods

n/a	Involved in the study	
☒	☐	ChIP-seq
☒	☐	Flow cytometry
☒	☐	MRI-based neuroimaging
